# The CD33 short isoform is a gain-of-function variant that enhances Aβ_1–42_ phagocytosis in microglia

**DOI:** 10.1186/s13024-021-00443-6

**Published:** 2021-03-25

**Authors:** Abhishek Bhattacherjee, Jaesoo Jung, Sameera Zia, Madelene Ho, Ghazaleh Eskandari-Sedighi, Chris D. St. Laurent, Kelli A. McCord, Arjun Bains, Gaurav Sidhu, Susmita Sarkar, Jason R. Plemel, Matthew S. Macauley

**Affiliations:** 1 Department of Chemistry, University of Alberta, 11227 Saskatchewan Dr., Gunning Lemieux Chemistry Centre E5-18A, Edmonton, T6G 2G2 Canada; 2 Neuroscience and Mental Health Institute, University of Alberta, Edmonton, T6G 2E1 Canada; 3 Department of Medicine, Division of Neurology, University of Alberta, Edmonton, T6G 2E1 Canada; 4 Department of Medical Microbiology and Immunology, Edmonton, T6G 2E1 Canada

**Keywords:** Alzheimer’s disease, CD33, Immunomodulatory, Isoform, Microglia, Phagocytosis, Mouse model, Gain-of-function

## Abstract

**Background:**

CD33 is genetically linked to Alzheimer’s disease (AD) susceptibility through differential expression of isoforms in microglia. The role of the human CD33 short isoform (hCD33m), preferentially encoded by an AD-protective *CD33* allele (rs12459419T), is unknown. Here, we test whether hCD33m represents a loss-of-function or gain-of-function variant.

**Methods:**

We have developed two models to test the role of hCD33m. The first is a new strain of transgenic mice expressing hCD33m in the microglial cell lineage. The second is U937 cells where the *CD33* gene was disrupted by CRISPR/Cas9 and complemented with different variants of hCD33. Primary microglia and U937 cells were tested in phagocytosis assays and single cell RNA sequencing (scRNAseq) was carried out on the primary microglia. Furthermore, a new monoclonal antibody was developed to detect hCD33m more efficiently.

**Results:**

In both primary microglia and U937 cells, we find that hCD33m enhances phagocytosis. This contrasts with the human CD33 long isoform (hCD33M) that represses phagocytosis, as previously demonstrated. As revealed by scRNAseq, hCD33m^+^ microglia are enriched in a cluster of cells defined by an upregulated expression and gene regulatory network of immediate early genes, which was further validated within microglia in situ. Using a new hCD33m-specific antibody enabled hCD33m expression to be examined, demonstrating a preference for an intracellular location. Moreover, this newly discovered gain-of-function role for hCD33m is dependent on its cytoplasmic signaling motifs, dominant over hCD33M, and not due to loss of glycan ligand binding.

**Conclusions:**

These results provide strong support that hCD33m represents a gain-of-function isoform and offers insight into what it may take to therapeutically capture the AD-protective *CD33* allele.

**Supplementary Information:**

The online version contains supplementary material available at 10.1186/s13024-021-00443-6.

## Background

Genome-wide association studies (GWAS) identified single nucleotide polymorphisms (SNPs) in *CD33* that correlate with AD susceptibility [1–4]. A meta–analysis of AD GWAS datasets has confirmed these findings [5]. The original SNP (rs3865444) was found within the *CD33* gene promoter but later discovered to be in linkage equilibrium with a second SNP (rs12459419) located within the second exon [6, 7]. The rarer rs12459419T allele correlates with decreased AD susceptibility and enhances exon 2 skipping [6, 7], leading to increased production of a short isoform known as human CD33m (hCD33m; m = minor) [8]. Conversely, the common rs12459419C allele correlates with increased AD susceptibility and favors production of a long isoform of CD33, known as human CD33M (hCD33M; M = major). To therapeutically modulate CD33 in a way that most effectively captures the AD protection revealed by genetic studies, a better understanding of the roles played by these two isoforms in microglia is needed. One unresolved question is whether hCD33m represents a loss-of-function variant stemming from decreased expression of hCD33M or whether increased expression of hCD33m has a gain-of-function.

CD33 is a member of the Sialic acid-binding immunoglobulin-type lectin (Siglec) family of immunomodulatory receptors [9, 10]. Many of the functions of Siglecs are regulated by interactions with glycan (carbohydrate) ligands via their N-terminal V-set domain [11], while inhibition of immune cell signaling is mediated by immunoreceptor tyrosine-based inhibitors motif (ITIM) and ITIM-like residues in their cytoplasmic domain [9]. The key difference between hCD33M and hCD33m is the absence of the V-set glycan-binding domain, which is encoded by exon 2, suggesting that glycan recognition could be involved in the correlation between hCD33 isoforms and AD susceptibility.

To date, the correlation between hCD33 alleles and AD susceptibility has been assessed by focusing on the immunomodulatory properties of hCD33 and its ability to regulate phagocytosis. Amyloid-β derived plaque accumulation is one of the early hallmarks of AD pathology, and many roles for microglia in modulating this process have emerged [12–14]. It was first demonstrated that hCD33M levels correlate with phagocytosis in primary monocytes [15]. Specifically, peripheral blood monocytes homozygous for the rare AD-protective *CD33* allele had increased phagocytosis relative to monocytes homozygous for the common AD-risk *CD33* allele. Notably, increased phagocytosis correlated with lower expression of hCD33M. However, these results do not differentiate between loss-of-function and gain-of-function roles for the two isoforms. Direct evidence for the loss-of-function hypothesis emerged from studies examining cultured mouse BV2 cells that overexpress hCD33M, which had impaired phagocytosis, while overexpression of hCD33m had no impact on phagocytosis [16]. Using a variety of human cell lines and transgenic mouse microglia expressing hCD33M, we confirmed that hCD33M represses phagocytosis [17]. More recently, a meta-analysis dataset [18] was reanalyzed [19], focusing on another *CD33* allele containing a four nucleotide (rs201074739) frameshift deletion before the transmembrane segment that prevents hCD33M from appearing on the cell surface [20]. Critically, it was found that rs201074739 does not correlate with AD susceptibility [19]. This suggests that the correlation between the rs12459419 alleles and AD susceptibility may not be solely due to a loss-of-function, stemming from decreased expression of hCD33M, but that there could be a gain-of-function role for hCD33m.

Testing a gain-of-function role for hCD33m has been hindered by three challenges: (i) human cells express both isoforms, making it difficult to attribute phenotypes to one isoform or the other; (ii) microglia are unique immune cells for which there are no cell culture models that faithfully mimic primary microglia; and (iii) differential splicing and *CD33* transcript is unique to humans [21]. Motivated to better understand the function of hCD33m in controlling microglia, we developed transgenic mice expressing hCD33m in the microglial cell lineage. Studying phagocytosis in microglia from these mice as well as performing single cell transcriptional profiling of these microglia has provided the first direct support for a gain-of-function role for hCD33m in the context of AD.

## Materials and methods

### Mice and animal care

All mice used in this study were made on a C57BL/6 background and further backcrossed onto a C57BL/6 J genetic background for a minimum of four generations. WT, CD45.1^+/+^ and Cx_3_cr1^Cre^ mice [22] were originally obtained from The Jackson Laboratory. Transgenic mice expressing hCD33M were described previously [23]. All animals used were maintained in an access-controlled barrier facility under specific-pathogen-free conditions. Studies were approved by the Heath Sciences Animal Care and Use Committee of the University of Alberta. Human blood samples were collected from healthy volunteers through an approved Institutional Human Research Ethics Board protocol.

### Generation of hCD33m transgenic mice

Rosa26-Stop^fl/fl^-hCD33m mice were generated on a C57BL/6 genetic background similar to the Rosa26-Stop^fl/fl^-hCD33M mice described previously [23], with minor modifications. Briefly, cDNA encoding hCD33m was cloned into the CTV targeting vector (Addgene) and validated by Sanger sequencing. Linearized plasmid was provided to The Centre for Phenogenomics for electroporation into C57BL/6NTac-C2 ES cells. Insertion of the targeted vector into the Rosa26 locus in ES cells was confirmed by PCR (Forward primer: 5′-GCAATAGCATCACAAATTTCAC-3′) and (Reverse primer: 5′-GCACACCGGCCTTATTC-3′) (band size 309 bp), which covers an overlapping region between the CTV vector and incorporated short isoform sequence. Two additional sets of primers helped verify the intact hCD33m gene: 5′-CACTCCTCGGTGCTCATAATC-3′ and 5′-GAAGATGAGGCAGAGACAAAGA-3′ (band size 261 bp); and 5′-CACTCCTCGGTGCTCATAATC-3′ and 5’TCCTCATCCATCTCCACAGTA-3′ (band size 434 bp). For these PCRs, the following program (95 °C = 2 min, 95 °C = 40s, 55 °C = 1 min, 72 °C = 2 min, 72 °C = 5 min with 35X cycle) and reagents (OneTaq® Hot Start 2X Master Mix with GC Buffer; New England Biolabs) were used. Following a blastocyst injection of one of the ES cell clones, chimeras were bred with C57BL/6 J albino mice [23, 24]. Rosa26-Stop^fl/fl^-hCD33m mice were genotyped by PCR using digested ear notch samples. A common forward primer located in the 5′ homology region (5′-GAGCTGCAGTGGAGTAGGCG-3′) was used. The WT Rosa26 locus was identified using a reverse primer (5′-TGCTGCATAAAACCCCAGAT-3′), with a band of 370 bp. Transgenic mice were detected using a reverse primer located in the CAG promoter (5′-GGGCGTACTTGGCATATGAT-3′), with a band of 566 bp [23].

### Mice expressing both the long and short isoform of hCD33

To generate mice expressing both hCD33M and hCD33m (*Cx*_*3*_*cr1*^*Cre+/−*^ *hCD33M*^*+/−*^ *hCD33m*^*+/−*^), we crossed *Cx*_*3*_*cr1*^*Cre+/+*^ *hCD33M*^*+/+*^ *hCD33m*^*−/−*^ mice with *Cx*_*3*_*cr1*^*Cre−/−*^ *hCD33M*^*−/−*^ *hCD33m*^*+/−*^ mice. Progeny containing hCD33m were genotyped by using forward primer (5′-GAGCTGCAGTGGAGTAGGCG-3′) and reverse primer (5′-TGCTGCATAAAACCCCAGAT-3′), and examined for the absence of a band at 370 bp.

### Mice expressing short isoform of hCD33 in absence of mouse CD33

To generate hCD33m transgenic mice on a mCD33^−/−^ background, mCD33^−/−^ mice were first crossed with hCD33m transgenic (*mCD33*^*+/+*^ *Cx*_*3*_*cr1*^*Cr+/−*^ *hCD33m*^*+/−*^) mice. Offspring that expressed hCD33m (*Cx*_*3*_*cr1*^*Cr+/−*^ *mCD33*^*+/−*^ *hCD33m*^*+/−*^) were selected and subsequently back-crossed with mCD33^−/−^ mice to generate the desired (*Cx*_*3*_*cr1*^*Cr+/−*^ *mCD33*^*−/−*^ *hCD33m*^*+/−*^) mice. Primary microglia isolated from *Cx*_*3*_*cr1*^*Cr+/−*^ *mCD33*^*−/−*^ *hCD33m*^*+/−*^ mice was used for competitive phagocytosis assay with non-transgenic mCD33^−/−^ as controls (*Cx*_*3*_*cr1*^*Cr+/−*^ *mCD33*^*−/−*^ *hCD33m*^*−/−*^).

### Flow cytometry

All flow cytometry data was collected on a 5-laser Fortessa X-20 (BD Bioscience) and analyzed using FlowJo software (BD Biosciences). Cell sorting took place on an Aria III (BD Bioscience).

### Intracellular staining

For U937 cells, cells were plated into a 96-well U-bottom plate and washed twice with PBS (300 rcf, 5 min, 4 °C), followed by treatment with trypsin at 37 °C for 10 min to remove the extracellular antigens. Cells were washed again twice with PBS (300 rcf, 4 °C) and labelled with Zombie Red™ Fixable Viability Dye in PBS (1:3000 dilution, Biolegend) at RT for 20 min in a dark room. After washing with PBS containing 5% FBS twice (300 rcf, 5 min, 4 °C), permeabilization and fixation were done together using a commercial Fixation/Permeabilization Solution Kit (BD Bioscience) on ice for 20 min. The fixed and permeabilized cells were washed twice with 1x BD Perm/Wash™ Buffer (2500 rcf, 5 min, 4 °C) and stained with either mIgG1-AF647 (0.5 μg/mL) or S503-AF647 (0.5 μg/mL) at 4 °C for 20 min. Before the flow assay, each well was washed with flow buffer two times (2500 rcf, 5 min, 4 °C) and transferred to flow tubes. For microglia staining, three brains from the same genotype were pooled together to isolate enough microglia. After plating the isolated microglia into a 96-well V bottom plate, the same steps were followed as above with U937 cells until after the wash with the 1x BD Perm/Wash™ buffer. Prior to staining with antibodies, cells were pre-incubated with TruStain FcX™ (anti-mouse CD16/21) antibody (Biolegend, 1:50 dilution) on ice for 10 min and the antibody cocktail was subsequently added to the microglia with the mouse Fc block solution and incubated on ice for an additional 30 min. The antibody cocktail included: CD11b (BV510, Biolegend, 1:200 dilution), Ly-6G (BV605, Biolegend, 1:500 dilution), Ly-6C (BV711, Biolegend, 1:500 dilution), CX_3_CR_1_ (PE, Biolegend, 1:200 dilution), CD45 (APC/Cy7, Biolegend, 1:200 dilution), F4/80 (BV421, Biolegend, 1:200 dilution) and anti-GFP (FITC, Biolegend,1:200 dilution), along with either mIgG1-AF647 isotype control or S503-AF647 at 1 μg/mL. After washing the samples twice with flow buffer (2500 rcf, 5 min, 4 °C), analysis was performed by flow cytometry.

### Isolation of adult mouse microglia and phagocytosis assay

Microglia were isolated from age (2–3 months old)- and sex-matched adult mouse brains according to our previously published protocol [17]. Briefly, the isolated microglia were washed (300 rcf, 5 min) and suspended in RPMI containing 10% FBS in preparation for the phagocytosis assay. To assess microglial phagocytosis in the most controlled manner, we performed a competitive phagocytic assay, similar to what we reported previously [17]. Briefly, hCD33M (*Cx*_*3*_*cr1*^*Cre+/−*^ *hCD33M*^*+/−*^) and hCD33m (*Cx*_*3*_*cr1*^*Cre+/−*^ *hCD33m*^*+/−*^) mice were directly tested against their WT counterparts (*Cx*_*3*_*cr1*^*Cre+/−*^ *hCD33M/m*^*−/−*^). To examine phagocytosis in microglia from transgenic mice expressing both short and long CD33 isoforms (*Cx*_*3*_*cr1*^*Cre+/−*^ *hCD33M*^*+/−*^ *hCD33m*^*+/−*^), we tested these in a competitive assay with microglia from CD45.1^+^ mice. The phagocytosis assay and preparation of the different fluorescent cargos were performed as described previously [17], with minor modifications. Briefly, microglial cells were plated in a V-bottom 96-well plate with 100 μl media containing 1:200 polystyrene beads (Fluospheres carboxylate-modified 1.0 μm blue 350/440, Molecular Probes, Life Technologies) or 400 nM aggregated amyloid-beta peptide (Aβ_1–42_, HiLyte Fluor 555-labeled, Anaspec Peptide, USA). The method for preparing the aggregated Aβ_1–42_ was adapted from a previous study where they have shown that this preparation yields aggregated/oligomers of Aβ_1–42_ [25]. Cells were incubated for 30 min at 37 °C. Following a wash, cells were stained with a cocktail of antibodies for 20 min at 4 °C that included: CD11b (APC/Cy7, clone M1/70, Biolegend, 1:200 dilution), Ly-6G (BV605, clone 1A8, Biolegend, 1:500 dilution), Ly-6C (BV711, clone HK 1.4, Biolegend, 1:500 dilution), Cx_3_cr1 (PerCP/Cy5.5, clone SA011F11, Biolegend, 1:200 dilution), and F4/80 (BUV395 clone T45–2342, BD Horizon, 1:200 dilution), CD45.1 (APC, clone A-20, Biolegend, 1:200), and CD45.2 (BV785, clone 104, Biolegend, 1:200. Following one wash, cells were suspended in flow buffer and analyzed by flow cytometry.

### Generation of microglia single cell suspensions for sequencing

Isolation and preparation of microglia for single cell gene expression studies were done according to a published protocol by Hammond et al. [26], with minor modifications. Briefly, mice were perfused with 30 mL of ice-cold HBSS buffer containing Actinomycin D (5 μg/mL) and Triptolide (10 μM). Isolated adult mice brains were stored in 10 mL storage buffer containing Actinomycin D (5 μg/mL), Anisomycin (27.1 μg/mL) and Triptolide (10 μM) at 4 °C. Brains were minced using a scalpel and were homogenized with a 5 ml syringe plunger in ice cold storage buffer through 40 μm filter (Corning) under sterile conditions. Cell suspensions were transferred to a pre-chilled 15 mL tube and centrifuged (300 rcf, 5 min, 4 °C). After centrifugation, the cell pellet was collected and was resuspended in 10 mL of ice cold 40% Percoll (Sigma) diluted in 1x (final) HBSS and centrifuged (30 min, 500 rcf, 4 °C). The microglia were pelleted following careful removal of the Percoll layer containing the myelin debris. The collected cell pellet was washed with 10 mL of ice-cold 1x HBSS buffer and centrifuged (5 min, 300 rcf, 4 °C). Samples were resuspended in 50 μl of ice-cold flow buffer (0.5% BSA, 1 mM EDTA, in 1x PBS, Sterile Filtered) containing antibodies targeting Cd11b (BV510, clone ICRF44, Biolegend, 1:200 dilution), CD45 (APC/Cy7, clone 30-F11, Biolegend, 1:200 dilution), and Cx3cr1 (PerCP/Cy5.5, clone SA011F11, Biolegend, 1:200 dilution) from Biolegend at a 1:200 dilution for 20 min on ice. After incubation, the samples were washed with ice cold flow buffer, centrifuged (5 min, 300 rcf, 4 °C) and resuspended in 700 μl of ice-cold cell sorting buffer (1x HBSS containing 10% FBS and 1 mM EDTA) in preparation for cell sorting. Approximately 70,000 microglia (CD11b^+^, CD45^+^, Cx_3_cr1^+^) were sorted using the 100 μm nozzle with a sort speed of approximately 3500 events/sec. Sorted samples were collected in 1.7 ml Eppendorf tubes. Sorted cells were centrifuged (300 rcf, 5 min, 4 °C) and the supernatant was removed. Pelleted cells were resuspended in 100 μL PBS + 0.1% BSA and immediately counted using a Neubauer chamber using 0.4% Trypan blue solution (Thermo Fisher). Samples with viability over 95% were used for experiments. Cells were resuspended in PBS in an appropriate volume to achieve a concentration of 1000 cells/mL. This cell suspension was used to generate the gel-beads + cell emulsion by the 10X Chromium Controller (PN-1000202) using the Chromium Next GEM Single Cell 3′ GEM, Library & Gel Bead Kit v3.1 (PN-1000121), Chromium Next GEM Chip G Single Cell Kit (PN-1000120) and Single Index Kit T Set A, (PN-1000213). Reverse transcription, cDNA amplification, library preparation, and sample barcoding were performed following the available manufacturer’s protocol. Finally, sample libraries were pooled and sequenced in Illumina HiSeq P150 (Sequencing type: Paired-end, single indexing) to an average depth of ~ 50,000 reads per cell.

### Single cell gene expression data analysis

The demultiplexed FASTQ files, generated from Illumina sequencing outputs, were aligned with the *Mus musculus* 10 (mm10) reference genome based on the STAR algorithm using the standard 10x Cell Ranger (v3.0.0) pipeline. Gene barcode matrices were generated for each sample using the unique molecular identifier (UMI) after filtering out non-specific barcodes. These matrices were imported into the R package Seurat (v3.21.) [27, 28] while retaining the cells with more than 200 genes and only the genes expressed in at least 3 cells, to account for empty droplets and sporadically expressed genes. We first removed low quality cells for each of the 3 datasets individually, removing all cells with more than 8000 genes (potential doublets/multiplets), mitochondrial count of greater than 10% (dying cells), and a minimum cell molecule count of 200. This resulted in the downsizing of the Control, hCD33M and hCD33m libraries from 6163, 5920, 6257 cells to 4455, 4195, 4835 cells, respectively (Experiment 1). In a second experiment (Experiment 2), Control, hCD33M and hCD33m libraries were filtered resulting in downsizing from an initial 3482, 5100, 5705 cells respectively to 3379, 4991, 5611 cells following quality control (Suppl. Fig. 7). We used the default scale when normalizing the gene expression and regressed out sex-specific genes (*Xist, Ddx3y, Eif2s3y, Erdr1, Gm29650, Kdm5d, Uty*) before running the principal component analysis (PCA). The ideal number of PCs (15) was determined using elbow plots and jack straw analyses, which we used for our initial clustering and UMAP projection.

Next, we converted the Seurat object to an AnnData object using a loom file as an intermediary, with LoomR and loompy allowing for its entry and exit into R and python environments, respectively. The loom file retained the Seurat UMAP embeddings, which we used to train the classifier in the Single-Cell Clustering Assignment Framework (SCCAF) machine learning model. SCCAF produces a confusion matrix that is then used to re-cluster single cell RNA sequencing data in an iterative manner [29]. After 150 iterations, we arrived at the final clustering display. We then used scanpy (single cell analysis in python) to rank the genes per cluster [30] and used recognized cell-specific genes in tandem with our calculated differentially expressed genes to annotate the cell types.

To assess gene regulatory networks by making use of the pySCENIC (v 0.10.0) (Single-Cell rEgulatory Network Inference and Clustering) pipeline with the *Mus musculus* 9 (mm9) reference genome. PySCENIC [31] determines the potential adjacencies between transcription factors and their downstream targets based on their co-expression via the GRNboost2 algorithm [32]. It uses the cisTarget databases to look at motif enrichment upstream of the target genes which refined the adjacencies matrix, removing the less likely targets and leaving the direct targets behind. The new established gene regulatory network of the transcription factor that includes the target genes and their regulators is termed a “regulon”. With SCENIC the level of regulon activity in each cell was scored using AUCell, which was then converted to a binary scale to reflect the presence or absence of the regulon the cells. The regulatory network activity was overlaid with the annotated SCCAF clustering and visualized using UMAP. We also isolated individual regulons of interest and visualized the networks using the iRegulon cytoscape plugin [33]. Finally, we used the top 50 differentially expressed genes per cluster to identify the associated pathways in gProfiler, using the gene ontology, KEGG and REACTOME databases.

### Generation of hCD33m-specific hybridomas

The gene encoding hCD33m (amino acids 1–132) was cloned into pcDNA5/FRT/V5-His-TOPO® vector (Thermo Fisher Scientific), which contained a C-terminal hIgG1-Fc, TEV cleavage site on the N-terminal side of the Fc, as well as a C-terminal His_6_ tag and a Strep-Tag II [34]. This construct was co-transfected with a plasmid pOG44, encoding the Flp recombinase, into Chinese Ovary Hamster (CHO) Flp-In cells using the manufacturer’s recommended protocol. Cells were grown in DMEM-F12 media containing 5% fetal bovine serum (FBS, Gibco), 1.0 mg/mL Hygromycin B, 100 U/mL of Penicillin & Streptomycin (P/S, Gibco) and 2.438 g/L Sodium Bicarbonate (Gibco). Selection with Hygromycin B (Thermo Fisher) took place for 10 days starting at 2 days post-transfection. For expression, one million hCD33m-Fc expressing CHO cells were added to a T175 flask with 50 mL of media (DMEM-F12, 5% FBS, 100 U/mL P/S, and 10 mM HEPES) and cultured for 12 days (10 days post-confluence). The supernatant was filtered through a sterile filtered unit (0.2 μm pore size, Fisher Scientific). The filtered supernatant was loaded into a 5 mL HisTrap excel column (GE healthcare) pre-equilibrated with 50 mL of equilibration buffer (20 mM Sodium Phosphate, 500 mM NaCl, pH 7.4). After washing the column with 75 mL of washing buffer (20 mM Sodium Phosphate, 500 mM NaCl, 20 mM Imidazole, pH 7.4), bound protein was eluted with 30 mL elution buffer (20 mM Sodium Phosphate, 500 mM NaCl, 500 mM Imidazole, pH 7.4) in 2.5 mL fractions. Eluted fractions containing hCD33m-Fc were pooled, diluted 10-fold in 20 mM sodium phosphate buffer (pH 7.2), and loaded into a Protein-G column (GE healthcare) pre-equilibrated with the same phosphate buffer. hCD33m-Fc was eluted with 0.1 M Glycine buffer (pH 2.7) directly in 1 mL fractions into microcentrifuge tubes containing 30 μL 1 M Tris-HCl (pH 8). The fractions containing protein were pooled and digested with 20 μL of 4 mg/mL His_6_-tagged TEV protease per 1 mL of 1 mg/mL protein solution at 37 °C for 3 h in a shaking incubator (150 rpm). The resultant reaction was put through a 1 mL HisTrap column to remove the bound Fc and TEV, while the hCD33m fragment came through in the flow through. The purified hCD33m was dialyzed three times into PBS and concentrated to 1 mg/mL using a 10 kDa cutoff Amicon ultra centrifugal filter unit (Millipore Sigma). In total, 4 mg of the CD33m fragment was prepared. An additional 2 mg of hCD33m-Fc was also saved for screening. The 4 mg of hCD33m and 2 mg of hCD33m-Fc were used for mouse immunization and monoclonal development (Thermo Fisher Scientific Pierce Proteomics). Supernatant from polyclonal hybridomas that were positive by ELISA for hCD33m-Fc were screened against CHO cells expressing hCD33M or hCD33m by flow cytometry using a PE-labelled anti-mouse IgG secondary antibody. Two hybridoma stocks uniquely stained hCD33m-expressing cells but not hCD33M-expressing cells. These two frozen hybridomas were thawed, expanded, and subcloned in RPMI media containing 20% FBS and 100 U/mL P/S by diluting to 1 cell per 3 wells in 96-well plates containing 220 μL of media. After 10 days, 50 μL of the supernatant from each well was screened against hCD33M-expressing, CD33m-expressing, and WT CHO cells by flow cytometry. A monoclonal hybridoma, designated S503, that produced antibody specific for hCD33m-expressing CHO cells was isolated.

### Expression, labelling, and isotyping of hCD33m-specific antibodies

For antibody expression, one million hybridoma cells were added to a T175 flask containing 50 mL of RPMI containing 10% FBS, 100 U/mL P/S, and 10 mM HEPES and cultured for five days after post-confluence. Each supernatant was filtered (0.2 μm pore size) and stored at 4 °C until ready for purification. Filtered supernatant (100 mL) was loaded onto a 1 mL Protein G column pre-equilibrated with 20 mM sodium phosphate buffer (pH 7.4). After washing the column with the 20 mM sodium phosphate buffer, anti-CD33m was eluted with 0.1 M glycine buffer (pH 2.7) in 1 mL fractions, directly into tubes containing 30 μL of 1 M Tris-base buffer (pH 8). The eluent was dialyzed three times against phosphate buffered saline (pH 7.4) and stored at 4 °C. For fluorophore labeling, antibody was concentrated to 0.2 mg/mL. Prior to fluorophore labelling, 50 μL of ammonium bicarbonate (1 M) was added to 950 μL of the 0.2 mg/ml solution of antibody solution to increase the pH, followed by the addition of 6.64 μL of 2.5 mM NHS-AF647 (Thermo Fisher Scientific), dissolved in DMSO, to achieve a 10-fold molar excess. The reaction was gently rocked for 1 h at 37 °C and immediately dialyzed three times in PBS. Mouse IgG1 isotype antibody (Thermo Fisher Scientific) was pre-dialyzed into the same PBS prior to labeling and then fluorophore labeled at the same time in order to achieve the same level of fluorophore modification. The ratio of absorption at A_280_ to A_647_ confirmed an antibody labelling ratio of appropriate 2.5 molecules of AF647 per antibody.

### Generation of CD33^−/−^ U937 cells

CRISPR/Cas9-mediated deletion of CD33 in U937 was carried out similarly as described previously [17], except a crRNA with the sequence CGGTGCTCATAATCACCCCA was used.

### Cloning of hCD33M and hCD33m into a RP172 vector

The two genes of hCD33M (accession number: NM_001772.4) and hCD33m (accession number: NM_001082618.2) were synthesized by GeneArt (Thermo Fisher). The C or T allele of hCD33 minigene DNA constructs were designed by including the two introns flanking exon 2 and placing a C or T at the 4th position of exon 2 for the rs12459419C and rs12459419T alleles (genes were synthesized by GeneArt). A 5′ NheI site and 3′ AgeI site were attached to each gene for ligation with a linearized RP172 vector with the same restriction enzyme combination. Subsequently, the genes were ligated into RP172 and transformed into NEB® competent *E. coli* cells. After isolating plasmids, the quality of each insert was identified through Sanger sequencing and RP172 containing either intact hCD33M or hCD33m gene was selected for viral transduction.

### Mutagenesis of the intracellular signaling residues

The ITIM (Y340 in hCD33M; Y213A in hCD33m) and ITIM-like (Y358 in hCD33M; Y231A in hCD33m) tyrosine residues were mutated to alanine through site directed mutagenesis with the following primers: ITIM-Forward, GGATGAGGAGCTGCATGCCGCTTCCCTCAACTTTC; ITIM-Reverse: GAAAGTTGAGGGAAGCGGCATGCAGCTCCTCATCC; and ITIM-like-Forward: CAAGGACACCTCCACCGAAGCCTCAGAGGTCAGGACCC, ITIM-like-Reverse: GGGTCCTGACCTCTGAGGCTTCGGTGGAGGTGTCCTTG). This mutagenesis took place in the pcDNA5/FRT/V5-His-TOPO vector. Each reaction mixture was digested by DpnI, and heat-inactivated at 65 °C for 15 min, and transformed into NEB® DH5훼 Competent *E. coli*, transferred to LB agar plates containing 100 휇g/mL ampicillin and incubated at 37 °C overnight. 6 colonies of each were selected and cultured in LB-Amp overnight. After miniprep, mutants were identified through Sanger sequencing.

### Lentivirus production

Lentivirus was produced and titered as previously described [17], with the following changes: 1 million HEK293T cells were plated in a 6-well plate on day 0. On Day 1, a transfection mix was made containing 625 ng RP18, 625 ng RP19, 1250 ng of transfer vector, 7.5 ul TransIT®-LT1 Reagent (Mirus Bio), and 258 μl Opti-MEM media (Gibco), and added to the HEK293T cells. 72 h post transfection, supernatant was harvested and concentrated using Lenti-X concentrator reagent (Takara Bio) as per manufacturer’s instructions.

### Lentiviral transduction of U937 cells

Lentivirus was titered as previously described [17]. 250,000 U937 cells were plated in a 12 well plate in 500 μl growth media. Lentivirus was added to each well at a MOI = 1 and incubated in a tissue culture incubator. 16 h post-transduction, media was topped up to 2 ml. 72 h following transduction, 300 μg/ml zeocin was added to each well as a selection agent to kill untransduced cells. Cells were monitored daily for cell death, and every 2 days the media was changed and re-supplemented with 300 μg/ml zeocin. After 1 week, cells were assayed on a flow cytometer to assess the purity of the selected population via the fluorescent marker mAmetrine. Cells were found to be > 97% mAmetrine positive after 1 week of zeocin selection. Phagocytosis assays were done 3 days after removing zeocin from the media.

### Phagocytosis assessment in U937 cells

U937 cells were grown up to ~ 1 × 10^6^ cells/mL density in a T75 flask and subsequently 100,000 cells were added to 96-well U-bottom plates in 100 μl of media/well. To initiate the phagocytosis assay, 100 μL of media containing fluorescent Aβ_1–42_ (400 nM) or polystyrene beads (1200 dilution from the vendor stock) was added to the cells and incubated for 30 min at 37 °C. After incubation period cells were washed with media twice and resuspended in flow buffer containing 1 μg/mL propidium iodide. Samples were analyzed by flow cytometry. Median fluorescence intensity (MFI) was used to assess extent of phagocytosis in Aβ_1–42_ treated samples while % of cells taking up at least one bead was counted for polystyrene beads. Cytochalasin-D (10 μM) pre-treated samples were also carried out in parallel and subtract for fluorescence not associated with phagocytosis.

### Analysis of CD33 expression on human primary monocytes

Human blood (3 mL) was incubated in 10 mL of red blood cell lysis buffer for five minutes and centrifuged (300 rcf, 5 min). The resulting pellet was lysed once to ensure that all red blood cells were removed. The remaining white blood cells were resuspended in Human TruStain FcX™ Fc receptor blocking agent (Biolegend; 1:100) in flow buffer for 10 min at room temperature. A solution of anti-human CD33 (Phycoerythrin, clone WM53, Biolegend, 1:250), antihuman CD14 (BV605, clone M5E2, BD horizon, 1:500), and anti-human CD15 (BUV395, clone HI98, BD Horizon, 1:500) antibodies were added to the cells. After incubation for 20 min on ice, cells were washed and resuspended in flow buffer containing 1 μg/mL propidium iodide.

### Immunofluorescence (IF) staining

Animals were anesthetized by isoflurane inhalation, perfused with approximately 30 mL of saline, the brain removed and cut in half, and each half was fixed in 4% phosphate-buffered paraformaldehyde (Thermo Scientific) at 4 °C a minimum of 24 h, followed by incubation with 30% sucrose (4 °C) for a minimum of 48 h. The brain was embedded in embedding medium for frozen tissue specimens (OCT, Thermo Scientific) and stored at − 80 °C until being further processed by cryostat. Coronal sections (20 μm) were allowed to warm to − 20 °C for at least 3 h prior to cryosectioning at − 20 °C. Cryosectioning was done within the hippocampus region, and for each brain a minimum of five sections were mounted onto each Superfrost Plus microscope slide (Thermo Scientific) and stored at − 80 °C.

For IF staining, slides were removed from − 80 °C, allowed to adjust to room temperature for 20 min and washed with PBS prior to permeabilization. Sample permeabilization was achieved by incubation with PBS containing 0.2% Triton-X100 (Bio-Rad) for 30 min, followed by 1 h blocking in 5% goat serum solution containing 0.05% Triton-X100. The slides were incubated with a minimal volume (200–400 μl) of 5% goat serum containing primary antibodies overnight at 4 °C. The following primary antibodies were used in our experiments: anti-NeuN (mouse monoclonal, Abcam, 1:300 dilution), anti-Iba-1 (rabbit monoclonal, FUJIFILM Wako Chemicals, 1:400 dilution) and anti-GFP (chicken polyclonal, Abcam; 1:400 dilution). The slides were washed three times in PBS-T (PBS containing 0.2% Tween-20) the following day and incubated with the secondary antibodies (AF488-conjugated anti-mouse, AF568-conjugated anti-rabbit, and AF647-conjugated anti-chicken, all used at 1:500 dilution) for 1 h, followed by three more washes in PBS-T. To minimize the fluorescent background, autofluorescence quenching kit (TrueVIEW, Vector Laboratories) was used as per the manufacturer’s protocol. Lastly, the slides were incubated with Hoechst (1:2000 dilution) for 15 min and cover-slipped with permanent mounting medium (TrueVIEW).

Fluorescence microscopy was performed with the LSM 700 laser scanning confocal microscope (ZEISS), equipped with Axiocam 702 mono camera (ZEISS). The images were captured at 20X magnification, and were analyzed with Zen2.6 Blue edition software (ZEISS). Total of five mice per group and a minimum of two brain sections from each animal were assessed for analysis.

### RNAscope analysis of in situ transcript levels

The RNAscope Fluorescent Multiplex Assay (ACD Biosystems) was performed according to the ACD protocol for fixed-frozen tissue according to the manufacturer’s instructions. In brief, hCD33m (*Cx*_*3*_*cr1*^*Cre+/−*^ *hCD33m*^*+/−*^) mice and WT controls (*Cx*_*3*_*cr1*^*Cre+/−*^ *hCD33M/m*^*−/−*^*)* were injected with terminal doses of Euthanyl before being euthanized via transcardial perfusion of PBS and 4% paraformaldehyde (PFA). Tissue was collected and fixed overnight with 4% PFA before cryoprotecting in a 30% sucrose solution, freezing, and cryosectioned (20 μm) onto slides. Brain sections from each mouse line were first pretreated with ethanol dehydration and target retrieval steps provided by ACDBio RNAscope, which includes a permeabilization step with a broad-spectrum protease for 30 min. Sections were then hybridized with 3 target-specific mRNA probes (*Fcrls*, *Fos and Klf2*) for 2 h at 40 °C, washed, then hybridized with amplifiers. The *Fcrls* RNAscope probe was used as the common universal microglial marker for each set of data. Additionally, the ACD 3-plex negative control probe was used on one parallel slide to confirm signal specificity. The probes were amplified according to manufacturer’s instructions and labeled with the following fluorophores for each experiment: OPAL Dyes 520, 570, and 620. All sections were stained with 4′,6-diamidino-2-phenylindole (DAPI). Images were of the hippocampus and cortex were acquired with a Leica TCS SP5 confocal microscope and images were counted for expression of *Fos* or *Klf2* within microglia, defined as *Fcrls* positive. Positive cells were defined as those with at least 3 RNAscope FISH mRNA probe signals located within the nucleus boundary (as indicated by DAPI) or 5 RNAscope FISH mRNA probes surrounding the nucleus.

### Statistical analyses

For experiments with only two groups, a two-tailed Student’s *t*-test was used to access statistical significance. When experiments were carried out in competition, a paired Student’s *t*-test was used, while an unpaired Student’s *t*-test was used in cases where experiments groups were analyzed independently. For experiments with more than two groups, a one-way ANOVA was carried out to determine if there were differences between the means. If applicable, the Tukey multiple comparisons post-test was then used to indicate significance between groups. N.S. means no statistical significance (*P* > 0.05).

## Results

### Generation of hCD33m transgenic mice and testing phagocytosis in hCD33m^+^ microglia

**Fig. 1:**
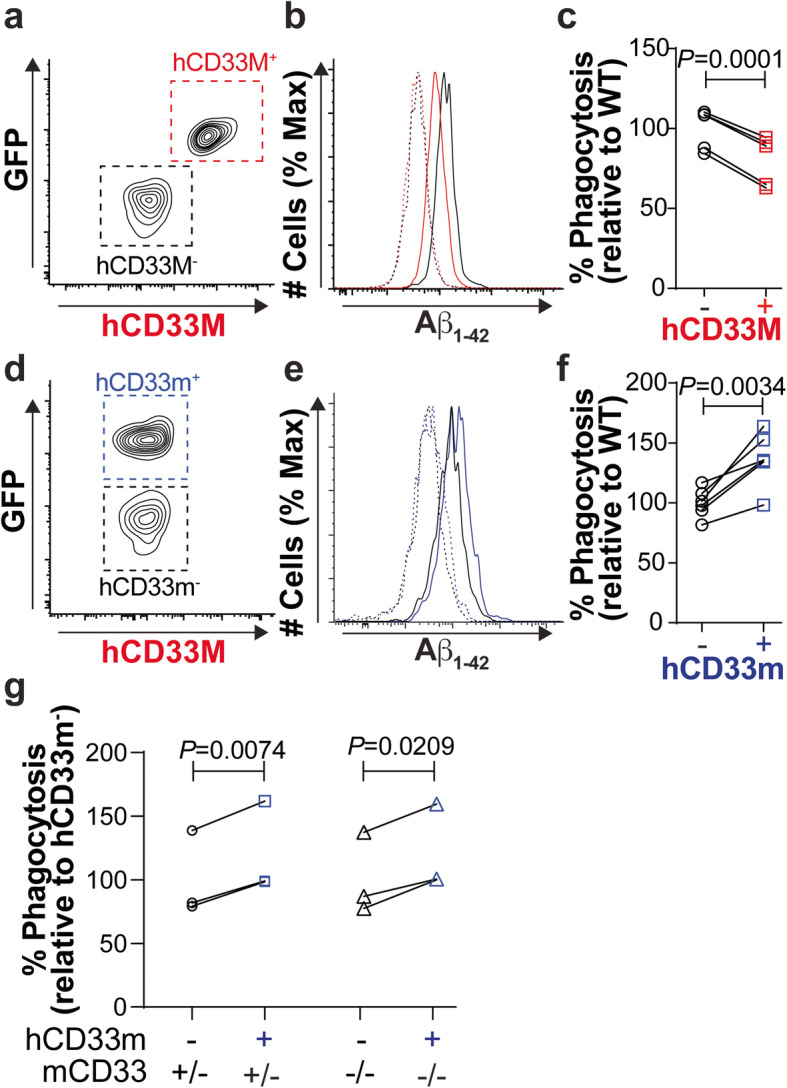
A gain-of-function revealed for mouse primary microglia expressing hCD33m. **a-c** A competitive flow cytometry-based phagocytosis assay between primary hCD33M^+^ (red) and WT (black) microglia, showing the (**a**) gating strategy, (**b**) representative plots for the uptake of aggregated fluorescent Aβ_1–42_ in the absence (solid line) or presence (dashed line) of cytochalasin-D by WT (black) and hCD33M^+^ (red) microglia, and (**c**) quantification of the MFI . % Phagocytosis represents the cytochalasin-D subtracted MFI values referenced to the average of the WT microglia set to 100%. (*N* = 5) (**d-f**) A competitive flow cytometry-based phagocytosis assay between primary hCD33m^+^ (blue) and WT (black) microglia, showing the (**d**) gating strategy, (**e**) representative plots for uptake of aggregated fluorescent Aβ_1–42_ in the absence (solid line) or presence (dashed line) of cytochalasin-D by WT (black) and hCD33m^+^ (blue) microglia, and (**f**) quantification of the MFI. % Phagocytosis represents the cytochalasin-D subtracted MFI values referenced to the average of the WT microglia set to 100%. (*N* = 6) (**g**) Results from a competitive flow cytometry-based phagocytosis assay between primary hCD33m^+^ (blue) and WT (black) microglia on a mCD33^−/+^ and mCD33^−/−^ background. % Phagocytosis represents the cytochalasin-D subtracted MFI values referenced to the average of the hCD33m^−^ microglia set to 100%. (*N* = 3)

Rosa26-Stop^flx/flx^-hCD33m mice were generated through stable transfection of mouse ES cells with the cDNA encoding hCD33m using the same method used previously for the generation of hCD33M transgenic mice [23]. Expression of hCD33m was driven in the microglial cell lineage by crossing Rosa26-Stop^flx/flx^-hCD33m mice with Cx3cr1^Cre^ mice. Brain sections from two-month old hCD33 transgenic mice revealed that the densities of microglia were not altered (Suppl. Fig. 1). Moreover, > 99.5% of the GFP^+^ cells, stemming from bicistronic expression of GFP, were found in the IBA-1^+^ cells, demonstrating minimal leakage into neurons or other cell types (Suppl. Fig. 2). Bicistronic expression of GFP enabled mixing of primary microglia isolated from adult hCD33M or hCD33m transgenic mice with age- and sex-matched WT microglia in a competitive phagocytic assay (Fig. 1a,d). Similar to our previously published data [17], hCD33M-expressing microglia phagocytosed significantly less aggregated fluorescently-labelled Aβ_1–42_ than WT microglia (Fig. 1b,c). Conversely, microglia from hCD33m transgenic mice phagocytosed significantly more aggregated Aβ_1–42_ compared to WT (Fig. 1e,f). A similar degree of enhanced phagocytosis within microglia from the hCD33m transgenic mice was observed with fluorescent polystyrene beads as cargo (Suppl. Fig. 3). We also assessed the effect of hCD33m on a mCD33^−/−^ background and found that expression of hCD33m increased phagocytosis to a similar extent on a mCD33^−/+^ versus mCD33^−/−^ background (Fig. 1g). These results demonstrate that hCD33M and hCD33m have opposing effects on phagocytosis, providing the first experimental evidence that hCD33m has a gain-of-function role in microglia.

### CD33 isoforms have opposing effects on phagocytosis in U937 cells

**Fig. 2:**
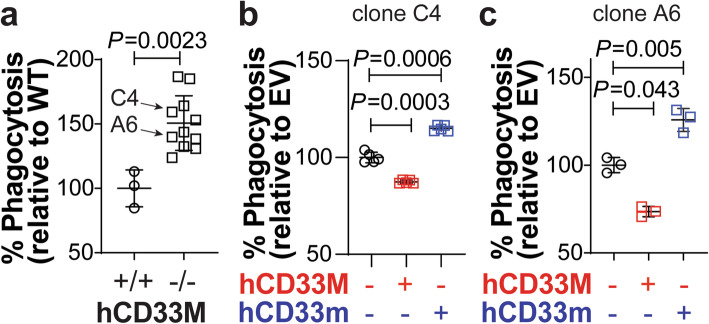
Differential regulation of phagocytosis by hCD33M and hCD33m in U937 cells. **a** Flow cytometric analysis of phagocytosis of aggregated fluorescent Aβ_1–42_ in hCD33^+/+^ and hCD33^−/−^ U937 clones. Levels of phagocytosis are standardized to the average of the three CD33^+/+^ set to 100%. Each data point represents one clone, which is itself the average of three replicates. Clones C4 and A6 are highlighted as they were used in subsequent transduction studies (N = 3 CD33^+/+^; *N* = 11 CD33^−/−^). % Phagocytosis represents the cytochalasin-D subtracted values referenced to the average of the CD33^+/+^ cells set to 100%. (N = 6) **b**,**c** hCD33M and hCD33m were transduced into CD33^−/−^ U937 cells (**b**) clone C4 and (**c**) clone A6, along with an empty virus control. Phagocytosis was assessed with aggregated fluorescent Aβ_1–42_ phagocytosis by flow cytometry. % Phagocytosis represents the cytochalasin-D subtracted values referenced to the average of the empty vector (EV) cells set to 100%. (B, *N* = 5 replicates; C, *N* = 3 replicates)

Previously, we demonstrated that CRISPR/Cas9-mediated deletion of CD33 in human U937 cells leads to increased phagocytosis, which can be complemented with lentivirus-mediated transduction of hCD33M [17]. However, complementation was not complete, with hCD33M repressing phagocytosis by only 5–10%. Aiming for more robust complementation, low passage CD33^−/−^ U937 clones were generated and verified to be, on average, 30–50% more phagocytic than CD33^+/+^ clones (Fig. 2a). Two of these CD33^−/−^ clones (clone C4 and A6) were transduced with lentivirus containing hCD33M, hCD33m, or empty vector (EV) control. Stably transduced cells were selected, and phagocytosis assessed with aggregated fluorescent Aβ_1–42_. In both clones, hCD33M-expressing cells displayed a 15–20% decrease in phagocytosis, demonstrating that complete complementation had been achieved (Fig. 2b,c). Conversely, hCD33m-expressing cells displayed a 10–15% increase in phagocytosis. These results support the opposing effects that hCD33M and hCD33m have on phagocytosis.

### Transcriptional profiling of microglia

**Fig. 3:**
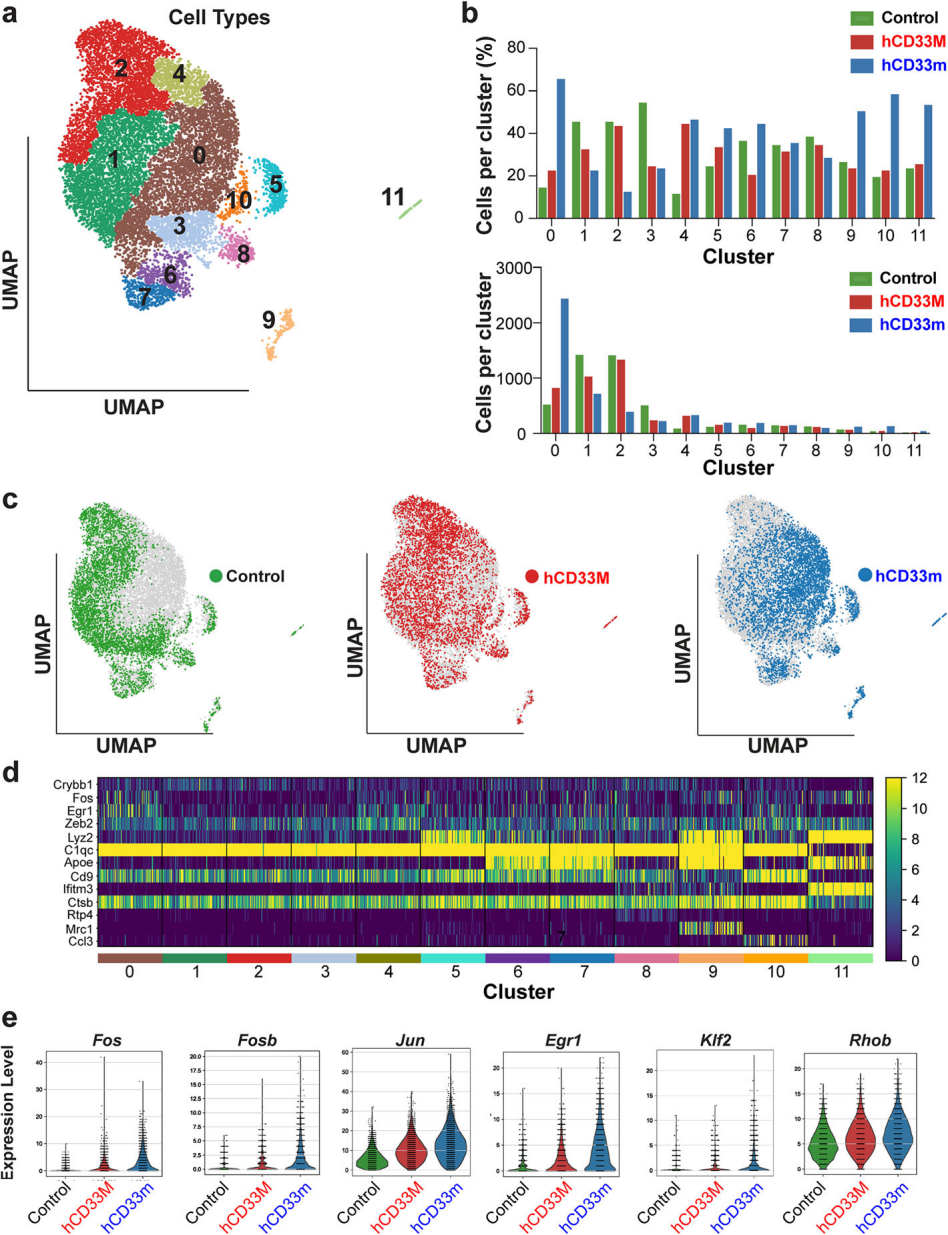
scRNAseq reveals transcriptional skewing in hCD33m microglia. **a** Unsupervised and iterative machine-learning based clustering using SCCAF of 13,485 cells in the merged analysis of control, hCD33m and hCD33m datasets from Experiment 1. A total of 12 clusters were identified. **b** Bar graphs showing the absolute number of cells from each isoform present in each cluster (top) and their respective proportions (bottom). **c** UMAP projection of the individual Control, hCD33M, and hCD33m datasets. **d** Heatmap demonstrating the cluster based differential expression of key stratifying genes. **e** Violin plots of genes from cluster 0. Expression levels in hCD33m^+^ microglia are significantly higher (*P* < 0.05) than the other two other genotypes for each gene

To investigate a basis for the gain-of-function role for hCD33m, single cell RNA sequencing (scRNAseq) was performed on primary microglia isolated directly from WT, hCD33M, and hCD33m mice. Two age-matched (two month) male mice from each genotype were perfused with cold PBS and the microglia were isolated from the brain under gentle conditions, making sure to keep the cells ice-cold throughout the entire procedure [35]. Isolated microglia were pooled for each genotype, labelled with antibodies, and the CD11b^+^CD45^low^Cx3Cr1^+^ cells sorted, as done previously [26]. Barcoded RNA libraries were generated and sequenced to an average depth of 50-80 K reads per cell. Stringent quality controls were used to exclude cells with higher microglial RNA content, leaving at least 4000 cells for each genotype. We initially clustered cells from the three pooled data sets with Seurat V3, before using single cell clustering assessment framework (SCCAF) to re-cluster cells. SCCAF uses an iterative machine learning approach to cluster cells that aligns well with expert-annotated scRNAseq data sets [29]. Clustered cells were projected onto a uniform manifold approximation and projection (UMAP) [36], which preserves organization of clustered cells (Fig. 3a). We identified twelve clusters in total: ten microglial cell clusters, one cluster of border associated microglia (BAM – Cluster 9) [37], and one monocyte cluster (Cluster 11); these latter two clusters may represent cells of the leptomeningeal, perivascular, or choroid plexus tissue from brain isolation. Microglia clusters were enriched with classic microglia markers such as *Sall1*, *Tmem119, and P2ry12*, while BAMs expressed *Ms4a7, Lyve1, and Mrc1,* and monocytes expressed *Ly6c2, Chil3, Plac8* (Suppl. Fig. 4) [37, 38]. By comparing where the cells from the individual genotypes map onto the clusters, it is readily apparent that hCD33m^+^ microglia are enriched within cluster 0 (Fig. 3a,b). In contrast, hCD33m^+^ microglia showed fewer cells in clusters 1 and 2, relative to the other two genotypes. Examining the percentage of cells in each cluster or proportion of each library in each cluster (Fig. 3c), it is apparent that > 50% of all hCD33m^+^ cells are in cluster 0. Examining the differentially expressed genes (DEGs) that define each cluster (Fig. 3d; Suppl. Fig. 5), we find that cluster 0 is characterized by upregulated expression of genes such as *Jun*, *Fos*, *Fosb*, *Erg1*, *Btg1*, *Btg2*, *Klf2*, *Rhob*, and *Zfp36l1* (Suppl. Fig. 6). Indeed, microglia isolated from hCD33m transgenic mice express significantly higher levels of *Fos*, *Fosb*, *Jun*, *Egr1*, *Klf2*, and *Rhob* compared to the other two genotypes (Fig. 3e). Conversely, clusters 1 and 2 are more characteristic of homeostatic microglia due to expression of *Csf1r*, *P2yr12*, *Ctsd*, *Cx3cr1*, *Cst3*, *Hexb*, and *Fcrls*. An independent scRNAseq experiment (Experiment 2) on the three different genotypes of mice was carried out and these same genes defining cluster 0 were found to be increased in hCD33m^+^ microglia (Suppl. Fig. 7).

We also identified fewer hCD33m^+^ cells in cluster 3 and more hCD33m^+^ cells in clusters 4, 9, 10 and 11. As mentioned, cluster 9 cells were enriched in BAM markers and cluster 11 cells were enriched with monocyte markers (Suppl. Fig. 4). Cluster 10 cells were sparse and enriched for the chemokine ccl4, suggestive of inflammatory activity (Suppl. Fig. 8). Fewer hCD33m^+^ and hCD33M^+^ cells were identified in cluster three, which was enriched for iron storage protein Ferritin Heavy Chain (*Fth1).* More hCD33m^+^ and hCD33M^+^cells were found in cluster 4 that were enriched in the cytoskeletal gene *Rock2.* Taken together, both hCD33m^+^ and hCD33M^+^ cells were associated with subtle shifts in gene expression for smaller populations of cells.

**Fig. 4:**
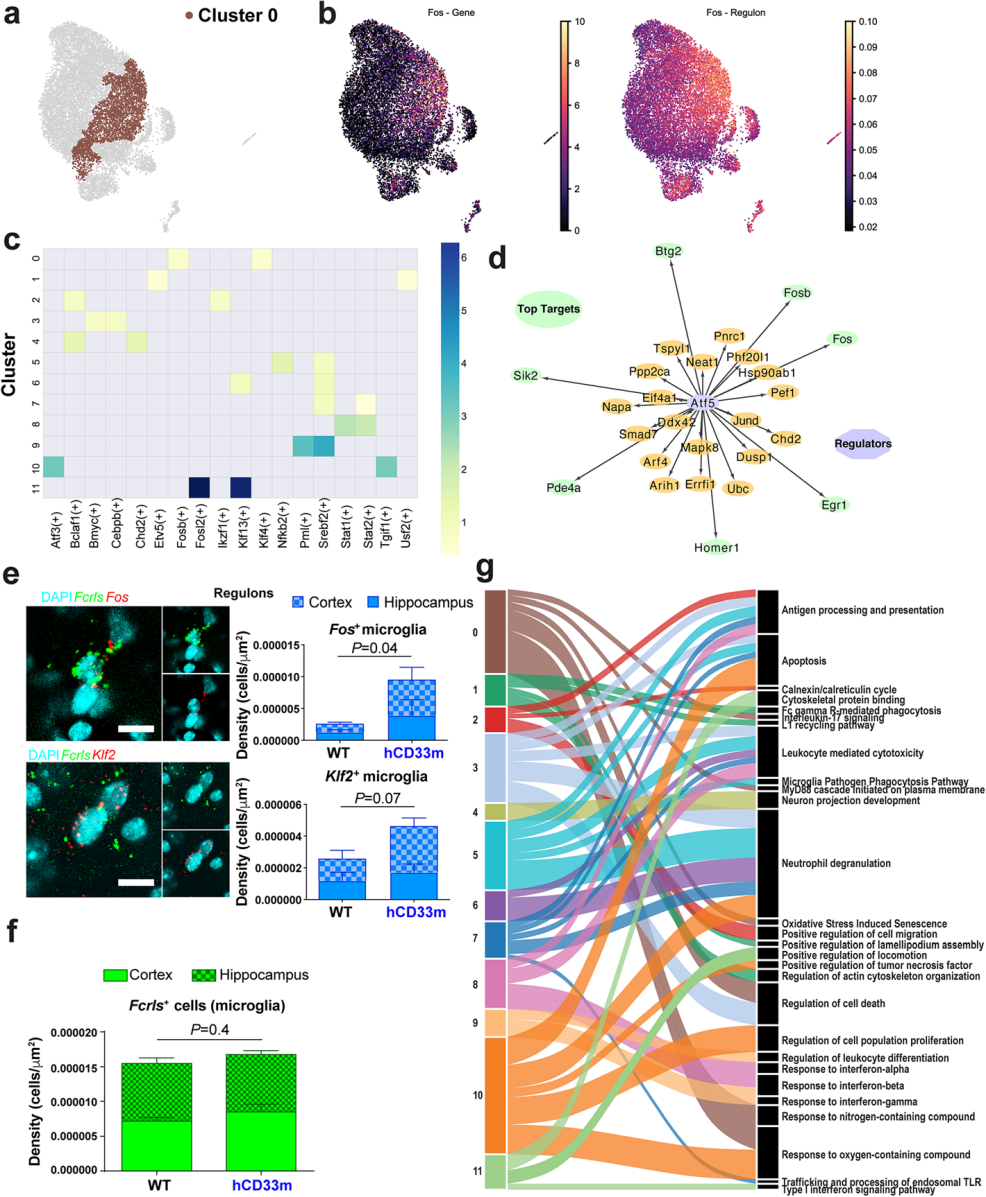
Pathway analysis reveals early-activating response elements upregulated in hCD33m microglia. **a** Cluster 0 from Experiment, a cluster enriched with immediate early genes such as *Fos*. **b** Comparison of the *Fos* gene single cell expression (left) and the entire Fos-regulon expression consisting of the dominating transcription factors, regulators and their downstream target genes. **c** SCENIC Heatmap of the major regulons in each identified cluster. **d** Schematic showing the *Atf5* regulator (purple) in the Fos-regulon and the target genes that it regulates (yellow), with the highly ranked/motif enriched targets in green. **e** Representative RNAscope images of cells positive for the microglia marker *Fcrls* (green), nuclear stain DAPI (cyan) and mRNA signals from either *Fos* (*upper*) or *Klf2* (*lower*) microglia subpopulations (red). Quantification of *Fos* and *Klf2* microglia cell density in the cortex and hippocampus of control and hCD33m^+^ isoform tissue. (*N* = 5 for each group) **f** Quantification of the number of microglia (*Fcrls*^*+*^) in brain WT and hCD33m^+^ mice. (*N* = 5 for each group) **g** Alluvial plot of the top pathways per cluster, band thickness corresponds to the number of genes associated with each pathway

Given that key transcription factors were upregulated in cluster 0, we wanted to determine gene regulatory network (GRN) changes within our single cell sequencing data. To assess the GRNs defining each cluster, we performed single-cell regulatory network interference and clustering (SCENIC) analysis [32] where we identified the regulons (transcription factors and their putative targets) with high expression levels at the single-cell level. SCENIC analysis demonstrated high expression of a *Fos*-dependent GRN predominantly in cluster 0. The *Fos* regulon enrichment expanded beyond cells expressing *Fos* suggesting that the *Fos* regulon, which encompasses *Fos* and its regulated elements, are ubiquitously located within a hCD33m enriched cluster (Fig. 4a,b). The *Fos* regulon was found associated with the *Fosb*, *Klf2*, and *Klf4* regulons, underscoring an unappreciated interaction with immediate early genes (IEGs; *Fos* and *Fosb), Kruppel Like factors (KLf)2*, and *Klf4* (Fig. 4c). A number of genes lie downstream of these regulators, of which *Egr1, Sik2, Btg2, Josd* rank highly and are accompanied by significant motif enrichment. The genes in the *Fos* regulon have been described as early activation response genes in microglia, playing a role in the recruitment of neutrophils through the secretion of IL1β and Cxcl1 (Fig. 4d) [39]. Nevertheless, these response elements have very recently been implicated as genes that can be upregulated ex vivo, particularly under conditions where the microglia are allowed to warm [35], meaning that they could potentially present an ex vivo artifact. Therefore, we performed *fluorescent* in situ *hybridization* with RNAscope to examine *Fos* and *Klf2* transcript levels in the brain of five age- and sex-matched WT and hCD33m^+^ mice in combination with *Fcrls* to identify microglia (Fig. 4e). We found that the density of *Fos*^*+*^ microglia are significantly increased with a trend for greater *Klf2*^*+*^ microglia density in the brain of hCD33m^+^ mice compared to WT mice (Fig. 4e). Importantly, there was no statistical difference in the number of microglia (*Fcrls*^*+*^) in the brain of hCD33m^+^ compared to a WT brain (Fig. 4f). These in situ results provide strong support that the transcriptional signature observed in hCD33m^+^ microglia do not arise ex vivo. To expand on GRN analyses we leveraged gene ontology, KEGG and REACTOME databases to define the functional breakdown of each cluster. The hCD33m^+^ enriched cluster 0 was defined by MyD88 and Il-17 signaling suggestive of a more activated state (Fig. 4g).

### Expression analysis of hCD33m through a newly developed hCD33m-specific antibody

**Fig. 5:**
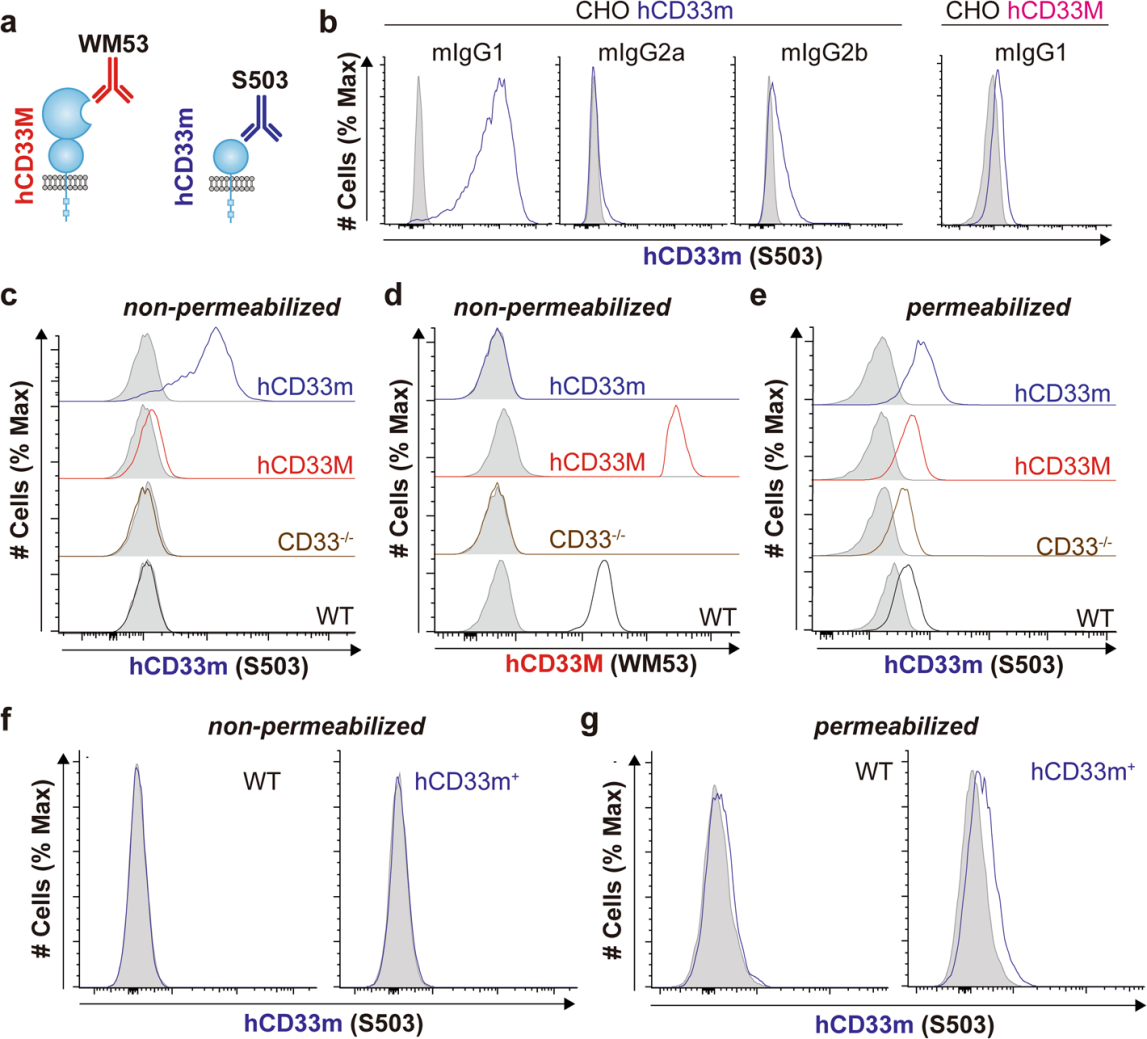
hCD33m drives a gain-of-function phenotype from an intracellular location. **a** Schematic demonstrating two anti-CD33 antibody clones. WM53 recognizes the N-terminal V-set domain unique to hCD33M while S503 recognizes a unique epitope on hCD33m. **b** S503 uniquely recognizes hCD33m-expressing CHO cells and is a mouse IgG1 antibody. Unstained S503 was incubated with cells CHO cells expressing hCD33m (left) or hCD33M (right), cell surface binding was detected with the indicated secondary antibody, and cells were analyzed by flow cytometry. **c**,**d** Cell surface expression of hCD33m and hCD33M with (**c**) S503 and (**d**) WM53, respectively, on WT or CD33^−/−^ U937 cells transduced with EV (CD33^−/−^), hCD33M, or hCD33m. **e** Intracellular staining of hCD33m in U937 cells from WT or CD33^−/−^ U937 cells transduced with EV (CD33^−/−^), hCD33M, or hCD33m. Cells were pre-blocked with unlabeled S503 followed by permeabilization of the cells prior to staining with AF647-labelled S503. **f**,**g** Expression of hCD33m from (**f**) non-permeabilized and (**g**) permeabilized primary microglia from WT and hCD33m^+^ mice

To examine expression of hCD33m, we developed a new hCD33m-specific monoclonal antibody, denoted as S503 (Fig. 5a). This antibody was raised against an immunogen comprised of the N-terminal extracellular domain of hCD33m lacking the transmembrane segment and cytoplasmic tail, made through an expression system that we recently described [34]. Clone S503 is a mouse IgG1 antibody, which strongly recognizes cell surface hCD33m, but not hCD33M, on transfected CHO cells (Fig. 5b). S503 was AF647-labelled and tested for binding to WT U937 cells as well as the CD33^−/−^ U937 cells (clone C4) transduced to express hCD33M, hCD33m, or neither (Fig. 5c). In extracellular staining, S503 bound strongly to cells overexpressing CD33m, showing no cell surface binding to the WT U937 cells and minimal binding to cells overexpressing hCD33M. Therefore, consistent with observations made by others [21, 40] our new S503 antibody does not detect endogenously-expressed hCD33m on the cell surface, except when overexpressed. Anti-hCD33 antibody clone WM53 recognizes the N-terminal V-set domain (Fig. 5a) and exclusively recognized CD33M-expressing cells as well as WT U937 cells (Fig. 5d). Anti-CD33 antibody clone HIM3–4, which was reported to recognize both isoforms of hCD33 [8, 21], showed a strong preference for hCD33M and minimal binding to hCD33m, with a small increase in binding following treatment of cells with neuraminidase (Suppl. Fig. 9).

Evidence has emerged that hCD33m is retained inside the cell [21, 41], therefore, we performed intracellular staining with S503 using a protocol that eliminates staining of extracellular antigen (Suppl Fig. 10a). Using this protocol, no difference in intracellular staining was observed in WT and CD33^−/−^ U937 cells, however, significant signal was observed in hCD33m-overexpressing cells (Fig. 5e, Suppl. Fig. 10b,c). Therefore, in U937 cells, S503 cannot detect endogenously expressed hCD33m but does detect intracellular hCD33m when overexpressed. Similar to U937 cells, endogenous expression of hCD33m could not be detected on the surface or within THP1 cells (Suppl. Fig. 10d).

Next, we carried out cell surface and intracellular staining with S503 on our CD33m transgenic primary mouse microglia. We could not detect expression of hCD33m on the surface of the hCD33m transgenic microglia (Fig. 5f). However, a small, reproducible signal relative to the isotype control was observed upon permeabilization in the hCD33m^+^ microglia (Fig. 5g). S503 did not detect signal on the surface of hCD33M^+^ microglia (Suppl. Fig. 11). Given that our hCD33m^+^ microglia only express hCD33m within an intracellular compartment, these results suggest that our transgenic mice do not represent an overexpressed system. Consistent with this, hCD33M expression levels on hCD33M^+^ transgenic mouse microglia show moderately lower expression levels compared to human primary peripheral blood monocytes (Suppl. Fig. 12). Moreover, at the transcript level, neither *hCD33* transgene was overexpressed compared to endogenous *mCd33* (Suppl. Fig. 13). Overall, the staining pattern of S503 supports the concept that hCD33m is retained within an intracellular compartment and that intracellular expression of hCD33m is sufficient to drive its gain-of-function phenotype in microglia.

### Probing the roles of signaling and glycan binding

**Fig. 6:**
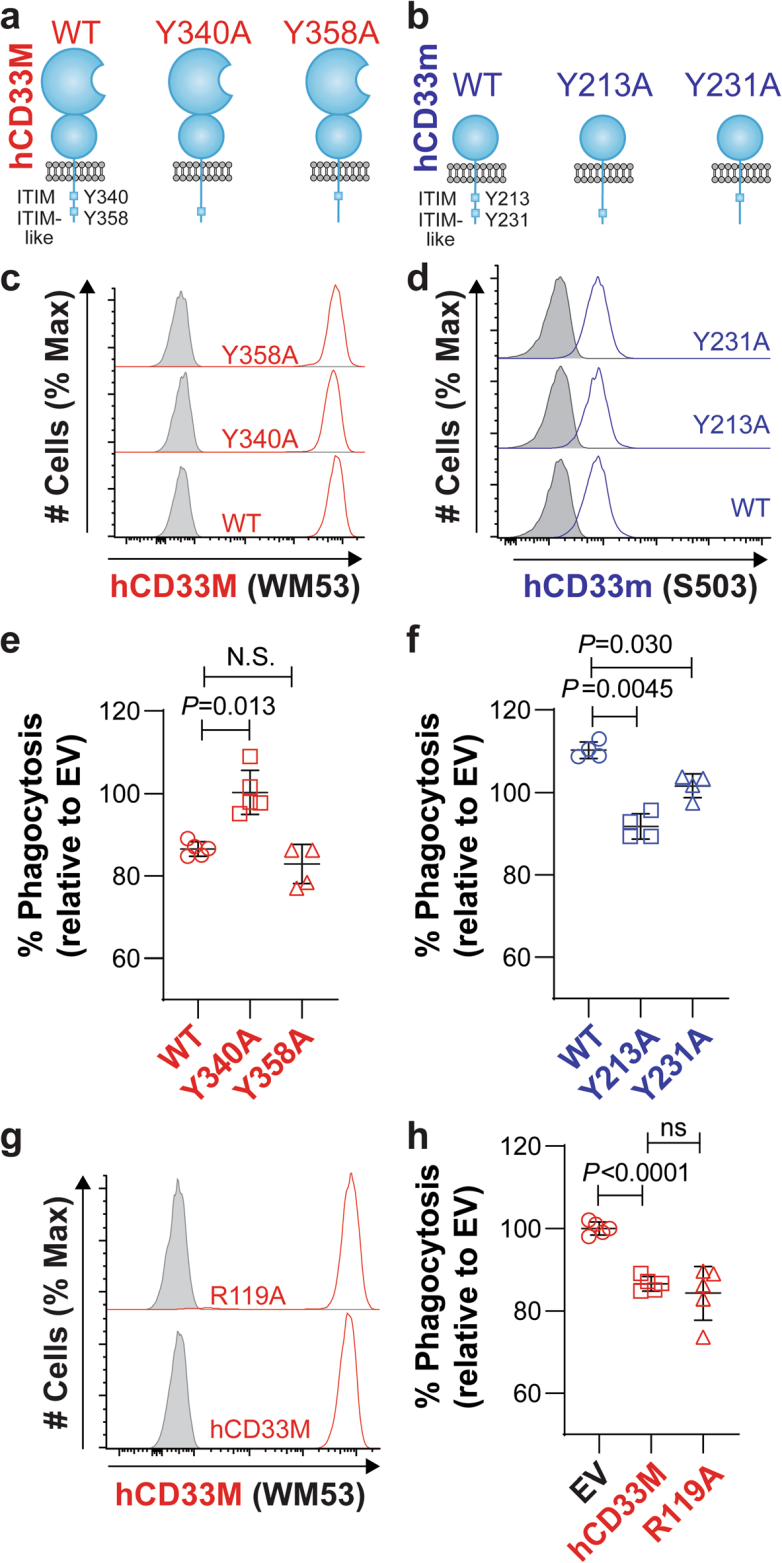
The gain-of-function role for hCD33m is signaling-dependent but not stem from loss of glycan-binding in hCD33M. **a**,**b** Cartoon diagram of the structure of (**a**) hCD33M and (**b**) hCD33m in the plasma membrane. The cytoplasmic tail of the proteins contains the ITIM and ITIM-like residues. Point mutants at each residue in each isoform were generated to test the importance of these residues. **c**,**d** Expression of (**c**) hCD33M (red) and (**c**) hCD33m (blue) and their associated mutants, transduced into CD33^−/−^ U937 cells. For hCD33M (**c**), cell surface expression (non-permeabilized) was assessed. For hCD33m (**d**), intracellular expression (permeabilized) was assessed. Isotype-stained cells are shown in grey. **e**,**f** Phagocytosis of aggregated fluorescent Aβ_1–42_ in CD33^−/−^ U937 (clone 4) cells virally-transduced to express (**e**) hCD33M or (**f**) hCD33m, along with their associated ITIM and ITIM-like mutants. % Phagocytosis represents the cytochalasin-D subtracted values referenced to the average of the empty vector (EV) cells set to 100%. (*N* = 4–5). **g** Cell surface expression of hCD33M and its R119A glycan-binding deficient mutant. Isotype-stained cells are shown in grey. **h** Phagocytosis of aggregated fluorescent Aβ_1–42_ in CD33^−/−^ U937 cells (clone 4) transduced to express WT or R119A hCD33M. % Phagocytosis represents the cytochalasin-D subtracted values referenced to the average of the empty vector (EV) cells set to 100%. (N = 5)

Siglecs regulate immune cell signaling from the cell surface through ITIMs located on their cytoplasmic tail [9]. To test a role for regulation of cellular signaling in the inhibitory and activatory role of hCD33M and hCD33m on phagocytosis, respectively, we generated point mutations for the ITIM (Y340 hCD33M; Y213A hCD33m) and ITIM-like (Y358M hCD33M; Y231A hCD33m) residues on both isoforms (Fig. 6a,b) and transduced these into the CD33^−/−^ U937 cells (clone C4) alongside their wild-type counterparts. These mutations did not perturb expression of either isoform (Fig. 6c,d). For cells expressing the hCD33M variants, we found that the ITIM is essential for the ability of hCD33M to suppress phagocytosis of aggregated Aβ_1–42_, whereas the ITIM-like residue is dispensable (Fig. 6e). For hCD33m, mutation of either residue led to a loss in the gain-of-function (Fig. 6f). Therefore, the gain-of-function role for hCD33m is dependent on these key signaling motifs.

To understand whether loss of glycan-binding has an effect on hCD33M-mediated repression of phagocytosis, a key conserved arginine residue (R119) in CD33, which is essential for glycan ligand recognition [11], was mutated to alanine in hCD33M. Reconstitution of CD33^−/−^ U937 cells (clone 4) with the hCD33M R119A mutant led to cell surface expression of this mutant at similar levels as WT hCD33M (Fig. 6g). Testing these cells in a phagocytic assay, we found that the R119A mutant repressed phagocytosis to the same extent as WT hCD33M (Fig. 6h). Therefore, an inability of hCD33M to interact with glycan ligands does not recapitulate observations associated with hCD33m, such as loss of cell surface expression and enhanced phagocytosis.

### Examining the gain-of-function role for hCD33m in the presence of hCD33M

**Fig. 7:**
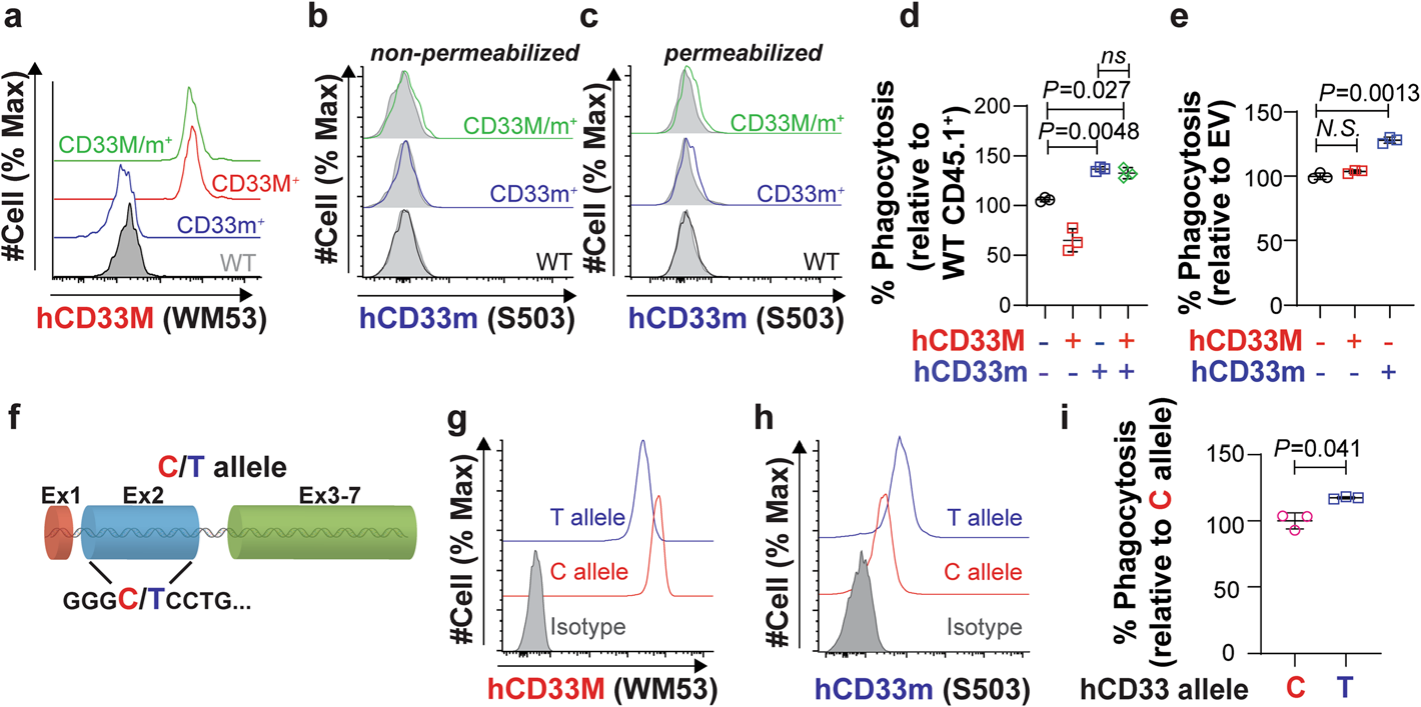
The gain-of function phenotype for hCD33m is dominant over the suppressive function of hCD33M. **a** Expression of hCD33M on primary microglia isolated from WT (gray), hCD33M^+^ (red), hCD33m^+^ (blue) and hCD33M^+^ hCD33m^+^ (green) mice. **b**,**c** Expression of hCD33m on primary microglia isolated from WT (black), hCD33m^+^ (blue) and hCD33M^+^ hCD33m^+^ (green) mice. Staining was carried out (**b**) without and (**c**) with permeabilization. Isotype-stained cells are shown in grey. **d** Phagocytosis of aggregated fluorescent Aβ_1–42_ in all four types of microglia: WT (blue), hCD33M^+^ (red), hCD33m^+^ (blue), and hCD33M^+^ hCD33m^+^ (green). Each cell type was tested in competition with WT CD45.1^+^ mice. Phagocytosis data is expressed as a relative percentage compared to WT CD45.1^+^ mice (*N* = 3). **e** Phagocytosis of aggregated fluorescent Aβ_1–42_ in WT U937 cells transduced with hCD33M (red), hCD33m (blue), or neither (EV) (black). Phagocytosis data is expressed as a relative percentage compared to EV (N = 3). **f** Schematic of a mini gene of CD33 containing only the two introns between Exon1 and 2 (intron 2) and Exon2 and 3 (intron 3). Two mini genes were created with a C or T at the corresponding location of the rs12459419 SNP. **g**,**h** Extracellular expression levels of (**g**) hCD33M and (**h**) hCD33m in CD33^−/−^ U937 cells transduced with the CD33 mini gene containing the C and T allele. Isotype-stained cells are shown in grey. **i** Phagocytosis of aggregated fluorescent Aβ_1–42_ in CD33^−/−^ U937 cells transduced with CD33 mini gene containing the C and T allele. Phagocytosis data is expressed as a relative percentage compared to C allele. (N = 3)

As human cells endogenously express both CD33 isoforms due to alternative splicing [8], we examined the effect of having both isoforms expressed in the same cells. In primary mouse microglia, this was accomplished by crossing our hCD33m and hCD33M transgenic mouse strains. Expression of both isoforms did not alter expression of hCD33M or hCD33m compared to cells expressing either isoform on its own, including hCD33m on the cell surface or within the cell (Fig. 7a-c). In a phagocytosis assay, microglia expressing both isoforms displayed increased phagocytosis to levels that were indistinguishable from microglia that express hCD33m alone (Fig. 7d). To probe this in cultured cells, WT U937 cells were used. Overexpression of hCD33M had no additional suppressive effect, while co-expression of hCD33m increased phagocytosis (Fig. 7e). These results suggest that the gain-of-function role of hCD33m is dominant over the suppressive effect of hCD33M.

**Fig. 8:**
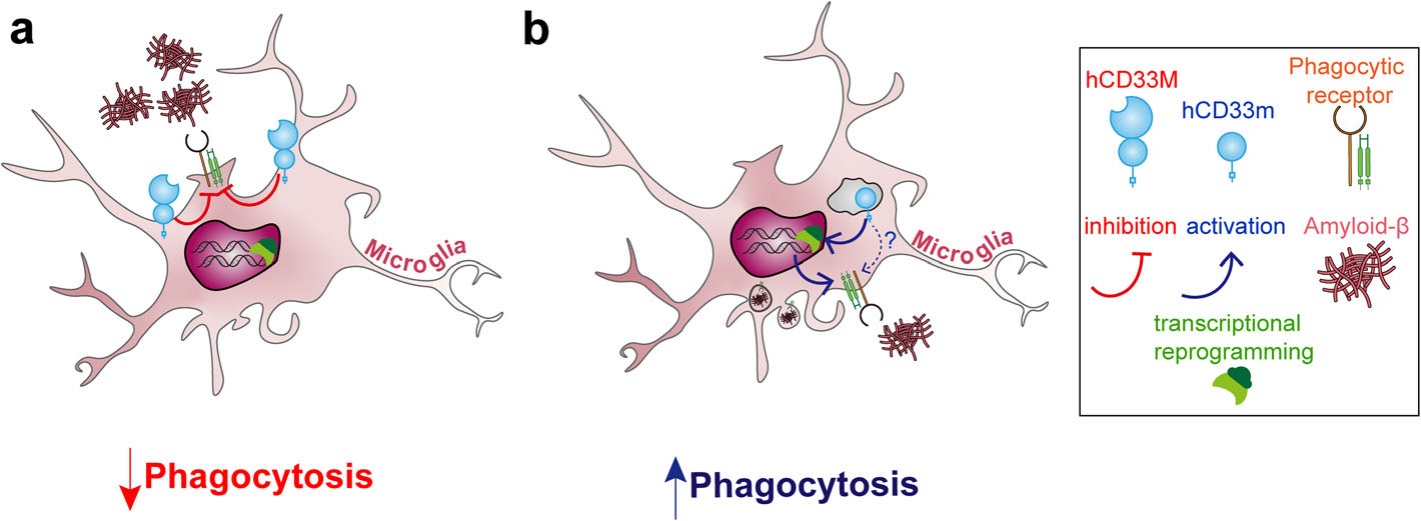
Model for the function of hCD33 isoforms in modulating phagocytosis. **a** hCD33M represses phagocytosis from the cell surface through its cytoplasmic ITIM motif. **b** hCD33m is located within an intracellular compartment and augments phagocytosis through transcriptional skewing

Finally, to examine the expression of hCD33M and hCD33m in the context of the rs12459419 allele, we created a *CD33* gene with the two introns flanking exon 2, which contained either a C or T at the rs12459419 location (Fig. 7f). These two genes were expressed in CD33^−/−^ U937 cells (clone C4) and the levels of hCD33M and hCD33m were assessed with WM53 (Fig. 7g) and S503 (Fig. 7h), respectively. The C allele expressed significantly higher levels of hCD33M and lower levels of hCD33m relative to the T allele. Moreover, cells expressing the T allele were more phagocytic (Fig. 7i). These results provide further support for a dominant gain-of-function phenotype induced by hCD33m. Taken together, our results suggest a model whereby intracellular hCD33m enhances phagocytosis, which correlates with transcriptional skewing in microglia, as opposed to hCD33M that antagonizes phagocytosis from the cell surface (Fig. 8).

## Discussion

Numerous genetic loci linked to AD susceptibility have converged on microglial cell phagocytosis [14]. Our results demonstrate that the short isoform of hCD33 is a gain-of-function variant that enhances microglial phagocytosis. Numerous groups have demonstrated that the rs12459419T allele undergoes alternative splicing to exclude exon 2, giving rise to elevated *hCD33m* transcript levels and a corresponding decrease in *hCD33M* transcript levels [6, 7, 42, 43]. Evidence for modulated expression of hCD33m protein has, however, been less definitive. Decreased protein levels of hCD33M was observed by Western blot and flow cytometry on peripheral blood monocytes and monocyte-derived microglia like cells [7, 44] as well as leukemic blasts from acute myeloid leukemia (AML) patients [42] homozygous for the AD-protective *CD33* SNP (rs3865444A/rs12459419T) compared to cells homozygous for the corresponding AD-susceptible *CD33* SNP. A corresponding increase in hCD33m protein levels has been more elusive, with a small increase observed in monocyte-derived microglia-like cells by Western blot [44], yet no differences seen in monocytes [7]. Although two other groups successfully raised a hCD33m-specific monoclonal antibody [21, 40], neither clarified whether the rs12459419T allele gives rise to enhanced hCD33m expression. Using our newly developed anti-hCD33m antibody (clone S503), we demonstrate that a *CD33* mini gene containing the introns flanks exon 2 with the corresponding rs12459419T allele leads to higher expression of hCD33m in U937 cells compared to the corresponding rs12459419C allele. Moreover, cells expressing the corresponding rs12459419T allele were more phagocytic relative to cells expressing the corresponding rs12459419C allele, re-enforcing the conclusion that the gain-of-function phenotype for hCD33m is dominant over hCD33M.

The enhanced phagocytosis observed in hCD33m^+^ microglia was associated with an elevation of IEGs by scRNAseq. This transcriptional skewing of hCD33m^+^ was observed in two independent scRNAseq experiments and by in situ hybridization. Others have shown an upregulation of these IEG, such as *Fos* and *Egr1*, to be preferentially enriched in samples that were dissociated under warm enzymatic conditions, suggesting that these genes are increased in microglia in response to environmental stimuli [35, 45]. Indeed, under homeostatic conditions, FACS-sorted microglia can have increased IEG expression compared to RiboTag methods where cells are not dissociated [46, 47]. We were careful to ensure that cells were kept cold throughout the entire isolation procedure, and microglia isolated from WT and hCD33M^+^ mice rarely associated with the *Fos*-microglia cluster. This raises the question of what are the functional consequences of IEG expression within hCD33m microglia? Little is known about the physiological or pathological role of IEGs within microglia. Jun and Fos belong to an activator protein family of transcription factors that are involved in numerous processes including: growth, immune responses, and cell death [48, 49]. Likely, these IEG are invoked by changes within the extracellular environment, such as axonal activity [50]. Therefore, we speculate that heightened IEG expression is indicative of a more vigilant or primed microglial state. Constitutive deletion of *Fosb* results in resistance to kainite excitotoxicity and associates with lower proinflammatory cytokines [51], suggesting that removal of IEG is essential for responding to environmental stimuli, albeit this study did not address whether these effects are due to microglial or neuronal *Fosb*.

Along with these IEGs, we observed altered expression of several notable genes linked to microglial proliferation and cytoskeleton remodeling, including *Rhob* and *Klf2*. These cellular processes are connected to phagocytosis in microglia [52, 53]. Upregulation of *Rhob* in hCD33m^+^ microglia is intriguing because its gene product, Rho-related GTPases binding protein (RhoB), stabilizes the active GTP-bound form of Rho, which in turn activates Rho kinase (ROCK), and is connected to cytoskeleton remodeling and microglial cell responsiveness [54]. Consistent with an essential role for ROCK in these processes, inhibition of ROCK in microglia impairs phagocytosis [55]. Furthermore, RhoB activation positively correlates with phagocytosis in human alveolar macrophages [56]. Upregulation of *Klf2* suggests that hCD33m^+^ may protect microglia from inflammatory insults because its gene product, Krüppel-like factor (Klf) 2, is a transcription factor associated with numerous protective roles in myeloid cells [57]. *Klf2*-deficient mice have exacerbated neurodegeneration [58] while *Klf2* overexpression or knockdown correlate positively with the response of monocytes to inflammatory conditions [59]. More closely connected with microglia, antagonistic neuroprotective anti-TREM2 antibodies stimulate enhanced microglial cell proliferation, and induce increased *Klf2* expression [60]. A direct protective role for Klf2 was also recently demonstrated in cultured mouse microglia [61].

Two pieces of evidence foreshadowed the gain-of-function for hCD33m that we report here. The first is the finding that hCD33m is retained inside the cell [21]. The second is that another allele of *CD33* (rs201074739), which disrupts cell surface expression of both isoforms, does not recapitulate the AD-protective effect of the rs12459419T allele [19]. These studies provided conceptual support that AD susceptibility connected to the rs12459419 loci may be more complex than loss-of-function stemming from decreased expression of hCD33M. The peroxisome localization remains intriguing because of the connection of this organelle to lipid metabolism, which is a process that becomes dysregulated in AD [62, 63]. Efforts to validate this localization are underway in our lab using our new hCD33m-specific antibody. We speculate that there is something about endogenously-expressed hCD33m that is not understood, which makes it difficult to detect. In our transgenic primary mouse microglia expressing hCD33m, no protein reached the cell surface, but evidence of intracellular accumulation was found, suggesting that the intracellular accumulation is sufficient to drive the gain-of-function. How is it then that the signaling motifs of hCD33m were required for the gain-of-function in U937 cells? It was previously speculated that hCD33m could play an activatory role through its ITIM and ITIM-like residues acting more like an Immunoreceptor Tyrosine-based Activatory Motif (ITAM) [19]. Consistent with this hypothesis, when either ITIM or ITIM-like were mutated on hCD33m, the gain-of-function phenotype was lost. This contrasted with hCD33M, where the ITIM played the dominant role; mutation of Y340 led to full loss of the repressive effect on phagocytosis while mutation of Y358 repressed phagocytosis to the same extent as WT hCD33M. More work is required to evaluate a positive signaling role for hCD33m and how this is connected to the transcriptional skewing. Interestingly, a very recent report studied the differences between CD33^−/−^ and CD33m-expressing induced pluripotent stem cell-derived microglia and found that the two genotypes produce different cellular responses, which is consistent with the alternative mechanisms we propose for CD33M and CD33m [64]. It is worth noting, however, that membrane-bound receptors are capable of modulating cellular signaling from within cellular compartments [65] and examples of modulation of cellular signaling from the peroxisome are emerging [66]. It should be cautioned that results carried out in U937 cells represent overexpressed conditions. Despite these overexpressed conditions, hCD33m and hCD33M produced the same effects on phagocytosis in U937 cells compared to more physiological levels within our primary transgenic mouse microglia.

Previously, we demonstrated that hCD33M represses phagocytosis [17], which was in line with previous observations [16]. Given that a key difference between hCD33M and hCD33m is the lack of the glycan binding domain in hCD33m, in our current study we tested whether glycan-binding by hCD33M plays a role in either: (i) the suppressive effect of hCD33M or (ii) the gain-of-function role for hCD33m. We found that a R119A mutant of CD33, which cannot bind its sialic acid-containing ligands [11], suppressed phagocytosis to a similar extent as WT hCD33M. Moreover, cell surface expression of the R119A mutant was not lost, unlike hCD33m that appears to be retained within an intracellular location. These findings do not rule out that glycan ligands of CD33 still contribute to modulating microglia within the brain through *trans* interactions with glycan ligands on other cells, as proposed for other Siglecs expressed on microglia [67]. A better understanding of the biochemical nature and location of CD33 ligands in the human brain may help address this possibility.

The results presented here have several important therapeutic implications. First, clinical trials are underway to address whether anti-CD33 antibodies (NCT03822208), which can deplete cell surface expression of hCD33M [6], can be beneficial in neurodegeneration. While our own results [17], including additional results in this work, confirm that genetic disruption of *CD33* enhances phagocytosis, it remains a distinct possibility that such a strategy will not capture the AD-protective gain-of-function role for hCD33m. In this regard, further studies into the mechanism of alternative splicing in the rs12459419T *CD33* allele may be warranted [43]. Another therapeutic implication for the gain-of-function role for hCD33m comes from studies that aim to genetically-manipulate CD33 in hematopoietic stem cells prior to bone marrow transplants. Several groups have demonstrated the feasibility of either deleting or manipulating the *CD33* gene within HSCs prior to a bone marrow transplant in AML patients to enable safer treatment with anti-CD33 therapy [68, 69]. One of these studies demonstrated that exon 2 could be deleted, through a CRISPR/Cas9 strategy that involves two guide RNAs targeting the introns flanking exon 2, to effectively generate hCD33m [70]. Given the gain-of-function role we have demonstrated here for hCD33m, we suggest that such an approach be used with caution until more is known about how hCD33m could be beneficial or detrimental in a physiological setting. Indeed, these results represent ex vivo studies of microglia expressing hCD33m and further investigation of the in vivo consequence of hCD33m is needed to better establish the link between increased hCD33m expression and AD protection. In this regard, the transgenic hCD33m-expressing mice generated in this study will serve as a useful tool in further evaluating the physiological roles of hCD33m in both health and disease. Specific to neurodegeneration, we also expect that these hCD33m transgenic mice will be valuable in examining the physiological significance of this gain-of-function role within mouse models of AD. Studies are underway to examine what impact hCD33M and hCD33m have on plaque accumulation in mouse models as well as skewing of microglia to disease-relevant subsets [71].

## Conclusions

In conclusion, our results in two different model systems provide strong evidence that hCD33m is a gain-of-function variant. This gain-of-function role was revealed by enhanced phagocytosis and transcriptional skewing in microglia expressing hCD33m. Taken together with the lack of AD protection afforded by the rs201074739 *CD33* deletion allele, these results support a model whereby the gain-of-function role for hCD33m plays a critical role in decreased AD susceptibility mediated by the rs12459419T *CD33* allele.

## Supplementary Information

**Additional file 1: Fig. 1**. Quantifying microglia in the brain of human CD33 transgenic mice by immunofluorescence staining. The density of microglia was assessed by the number of IBA1 + cells per mm2 of tissue from the average of a minimum of five sections from each genotype. The microglia density was evaluated in (a) whole brain, and in three selected regions: (b) cortex, (c) hippocampus, and (d) midbrain. No significant difference was detected amongst the four cohorts that were analyzed (*N* = 5 mice per genotype). **Fig. 2**. Quantifying GFP expression in the brain of human CD33 transgenic mice by immunofluorescence staining. (a) The percentage of GFP positive cells in the IBA1+ or NeuN+populations were assessed in a minimum of five individual sections from five mice per genotype. Representative images are shown for each genotype. (b,c) Quantifying the percentage of GFP + cells in the (b) IBA1+ and (c) NeuN+ reveals minimal leakiness outside of the microglial cell lineage. **Fig. 3**. hCD33m transgenic microglia have an enhanced ability to phagocytose fluorescent beads. A competitive flow cytometry-based phagocytosis assay between primary hCD33m+ (blue) and WT (black) microglia, showing the (a) representative flow cytometry data for uptake of fluorescent polystyrene beads and (b) quantification of uptake. % Phagocytosis represents the cytochalasin-D subtracted values referenced to the average of the WT microglia set to 100%. (N=6). **Fig. 4**. Cell annotation for BAMs, monocytes and microglia. (a) BAM specific gene markers, *Ms4a7, H2-Eb1* and *Mrc1* exclusively expressed in cluster 9. (b) *Ly6c2, Plac8* and *Ace*, genes representative of monocytes expressed in cluster 11. (c) Validation for the microglia specific genes, *Sall1, Tmem119* and *P2ry12* for clusters 0–8. **Fig. 5**. Differential gene expression of the microglia clusters from Experiment 1. (a) UMAP projection of the 12 identified clusters from Experiment 1. (b) Heatmap showing the top 50 differentially expressed genes in each cluster. **Fig. 6**. The top 30 DEGs in Cluster 0 from Experiment 1. **Fig. 7**. Single cell analysis of control, hCD33M and hCD33m in Experiment 2 reveals differences in isoform gene expression. (a) UMAP projections of the 13,982 cells in the merged Experiment 2 datasets showing 13 individual clusters. (b) Bar graphs showing the absolute number of cells from each isoform present in each cluster (top) and their respective proportions (bottom). (c) UMAP projection of the individual Control, hCD33M and hCD33m datasets. (d) Heatmap of representative genes. (e) Violin plots of hCD33m specific cluster 0 genes. **Fig. 8**. Feature plots showing the differentially expressed genes for each of the 11 lusters. Cluster were defined by the unsupervised SCCAF clustering and the expression of two representative genes were chosen for each cluster. Cluster 0–8, and 10 expressed microglial genes, whereas cluster 9 expressed border associated macrophage genes and cluster 11 expressed monocyte genes. **Fig. 9**. Anti-CD33 clone HIM3–4 does not recognize hCD33m. U937 cells overexpressing either hCD33M or hCD33m tested with anti-CD33 antibody clone HIM3–4 before and after pre-treatment with neuraminidase. **Fig. 10**. Optimizing and quantifying intracellular staining with S503 on U937 and THP1 cells. (a) CD33−/− U937 cells overexpressing CD33m were used to optimize a procedure with trypsin to remove cell surface antigens. Cells were treated with or without trypsin prior to staining with S503 (blue) or isotype control (grey). Cells were not fixed or permeabilized in this experiment. (b) Quantification of the mean fluorescence intensity (MFI) values for S503 staining of U937 cells with the indicated genotypes, taken from Fig. 5e of the main manuscript. MFI values are isotype control-subtracted. (c) An independent experiment showing that intracellular staining of hCD33m can be detected within hCD33m-overexpressing CD33−/− U937 cells. (d) Extracellular (*left panel*) and intracellular (*right panel*) staining with S503 and isotype control (grey) on WT and CD33−/− THP1 cells. **Fig. 11**. Cell surface staining of hCD33m + and hCD33M+ transgenic primary mouse microglia with antibody S503. (a) Flow cytometry histograms of WT (black), hCD33M+ (red), and hCD33m + (blue) primary microglia stanine with either isotype or S503. (b) Quantification of the mean fluorescence intensity (MFI) values for *n* = 3 samples for each condition. *N.S.* = no statistical significance (*P* > 0.05). **Fig. 12**. hCD33M+ mouse microglia express hCD33M at levels modestly lower than human peripheral blood monocytes. (a) Peripheral blood mononuclear cells were assessed for CD33M expression (red) on CD14+ peripheral blood monocytes relative to an isotype control (grey). (b) hCD33M+ primary mouse microglia were assessed for CD33M expression (red) relative to an isotype control (grey). (c) Mean fluorescence intensity (MFI) values from the flow cytometry graphs of CD33M signal. Data represents three different healthy human subjects and three different CD33M+ mice. **Fig. 13**. CD33 transcript levels in transgenic mouse microglia. (a) *mCd33* transcript levels in primary microglia from hCD33 transgenic mice demonstrate that expression of neither hCD33 isoform alters the *mCd33* transcript levels. (b) *hCD33* transcript levels in primary microglia from hCD33 transgenic mice. Both datasets are derived from aligning our scRNAseq datasets with the inclusion of *hCD33*. Note that this method does not differentiate *hCD33M* and *hCD33m* transcripts due to extensive overlap between the two isoforms.

## Data Availability

The RNA-seq expression data has been deposited to the GEO database.

## Abbreviations

AD: Alzheimer’s disease
hCD33M: Human CD33 major isoform
hCD33m: Human CD33 minor isoform
GWAS: Genome-wide association studies
SNP: Single nucleotide polymorphism
Siglec: Sialic acid-binding immunoglobulin-type lectin
SCCAF: Single-Cell Clustering Assignment Framework
pySCENIC: Python Single-Cell rEgulatory Network Inference and Clustering
GFP: green fluorescent protein
CRISPR: Clustered regularly interspaced short palindromic repeats
UMAP: Uniform manifold approximation and projection
IEG: Immediate early gene
CHO: Chinese hamster ovary
scRNAseq: Single cell RNA sequence
EV: Empty vector
BAM: Border associated microglia
DEGs: Differentially expressed genes
GRN: Gene regulatory network
ITIM: Immunoreceptor tyrosine-based inhibitory motif
ITAM: Immunoreceptor tyrosine-based activatory motif
AML: Acute myeloid leukemia
