# Supplementary material for: The CD33 short isoform is a gain-of-function variant that enhances Aβ1–42 phagocytosis in microglia
Source: Mol Neurodegener. 2021 Mar 25;16:19. doi: 10.1186/s13024-021-00443-6 (PMC7992807; doi:10.1186/s13024-021-00443-6)
Supplement: Supplementary file 1 — Additional file 1: Fig. 1. Quantifying microglia in the brain of human CD33 transgenic mice by immunofluorescence staining. The density of microglia was assessed by the number of IBA1 + cells per mm2 of tissue from the average of a minimum of five sections from each genotype. The microglia density was evaluated in (a) whole brain, and in three selected regions: (b) cortex, (c) hippocampus, and (d) midbrain. No significant difference was detected amongst the four cohorts that were analyzed (N = 5 mice per genotype). Fig. 2. Quantifying GFP expression in the brain of human CD33 transgenic mice by immunofluorescence staining. (a) The percentage of GFP positive cells in the IBA1+ or NeuN+populations were assessed in a minimum of five individual sections from five mice per genotype. Representative images are shown for each genotype. (b,c) Quantifying the percentage of GFP + cells in the (b) IBA1+ and (c) NeuN+ reveals minimal leakiness outside of the microglial cell lineage. Fig. 3. hCD33m transgenic microglia have an enhanced ability to phagocytose fluorescent beads. A competitive flow cytometry-based phagocytosis assay between primary hCD33m+ (blue) and WT (black) microglia, showing the (a) representative flow cytometry data for uptake of fluorescent polystyrene beads and (b) quantification of uptake. % Phagocytosis represents the cytochalasin-D subtracted values referenced to the average of the WT microglia set to 100%. (N=6). Fig. 4. Cell annotation for BAMs, monocytes and microglia. (a) BAM specific gene markers, Ms4a7, H2-Eb1 and Mrc1 exclusively expressed in cluster 9. (b) Ly6c2, Plac8 and Ace, genes representative of monocytes expressed in cluster 11. (c) Validation for the microglia specific genes, Sall1, Tmem119 and P2ry12 for clusters 0–8. Fig. 5. Differential gene expression of the microglia clusters from Experiment 1. (a) UMAP projection of the 12 identified clusters from Experiment 1. (b) Heatmap showing the top 50 differentially expressed genes in each clu [file 13024_2021_443_MOESM1_ESM.pdf]

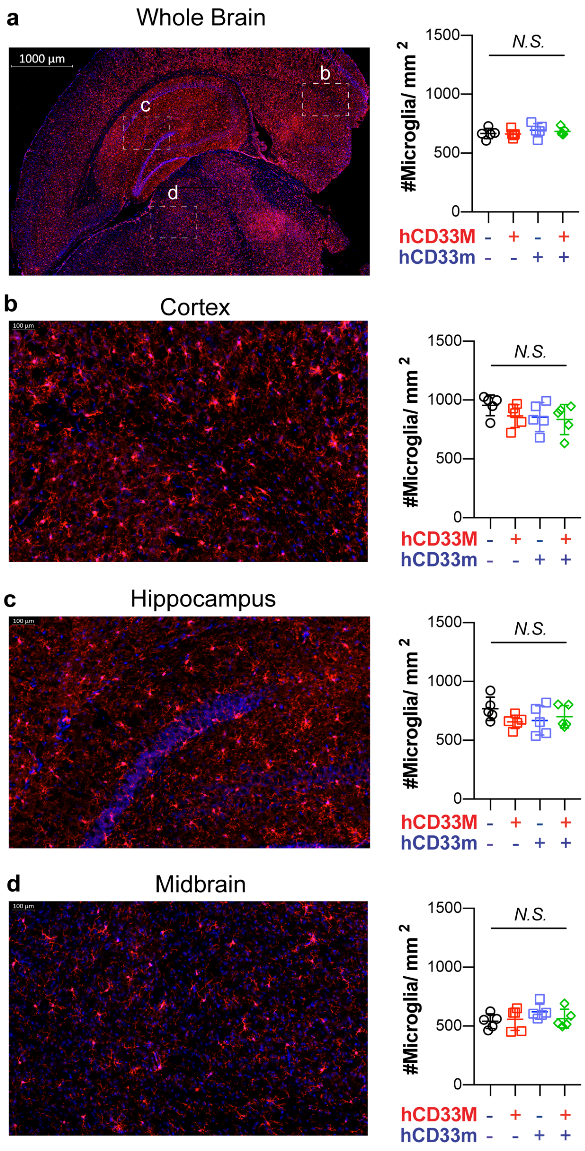

Suppl Fig. 1. Quantifying microglia in the brain of human CD33 transgenic mice by immunofluorescence staining. The density of microglia was assessed by the number of IBA1$^+$ cells per mm^2 of tissue from the average of a minimum of five sections from each genotype. The microglia density was evaluated in (**a**) whole brain, and in three selected regions: (**b**) cortex, (**c**) hippocampus, and (**d**) midbrain. No significant difference was detected amongst the four cohorts that were analyzed (N=5 mice per genotype).

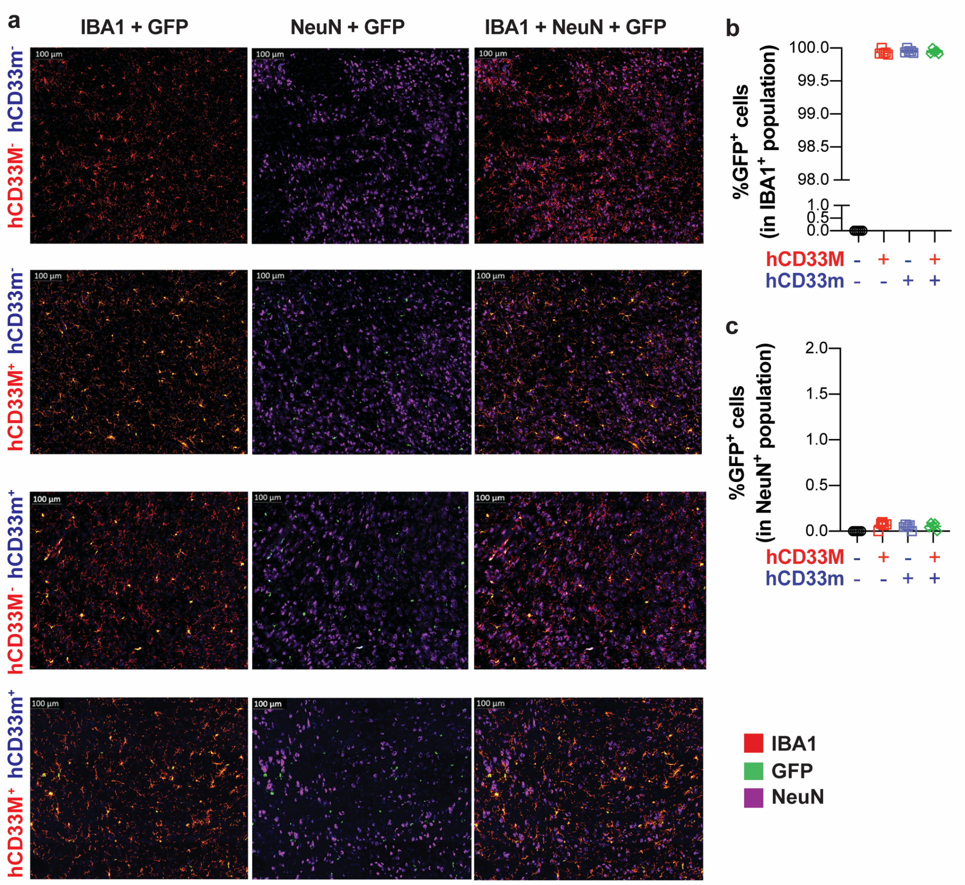

Suppl Fig. 2. Quantifying GFP expression in the brain of human CD33 transgenic mice by immunofluorescence staining. (a) The percentage of GFP positive cells in the IBA1^{+} or NeuN^{+} populations were assessed in a minimum of five individual sections from five mice per genotype. Representative images are shown for each genotype. (**b**,**c**) Quantifying the percentage of GFP^{+} cells in the (**b**) IBA1^{+} and (**c**) NeuN^{+} reveals minimal leakiness outside of the microglial cell lineage.

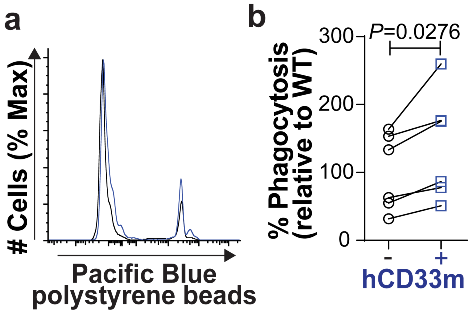

Suppl Fig. 3. hCD33m transgenic microglia have an enhanced ability to phagocytose fluorescent beads. A competitive flow cytometry-based phagocytosis assay between primary hCD33m^{+} (blue) and WT (black) microglia, showing the (**a**) representative flow cytometry data for uptake of fluorescent polystyrene beads and (**b**) quantification of uptake. % Phagocytosis represents the cytochalasin-D subtracted values referenced to the average of the WT microglia set to 100%. (N=6)

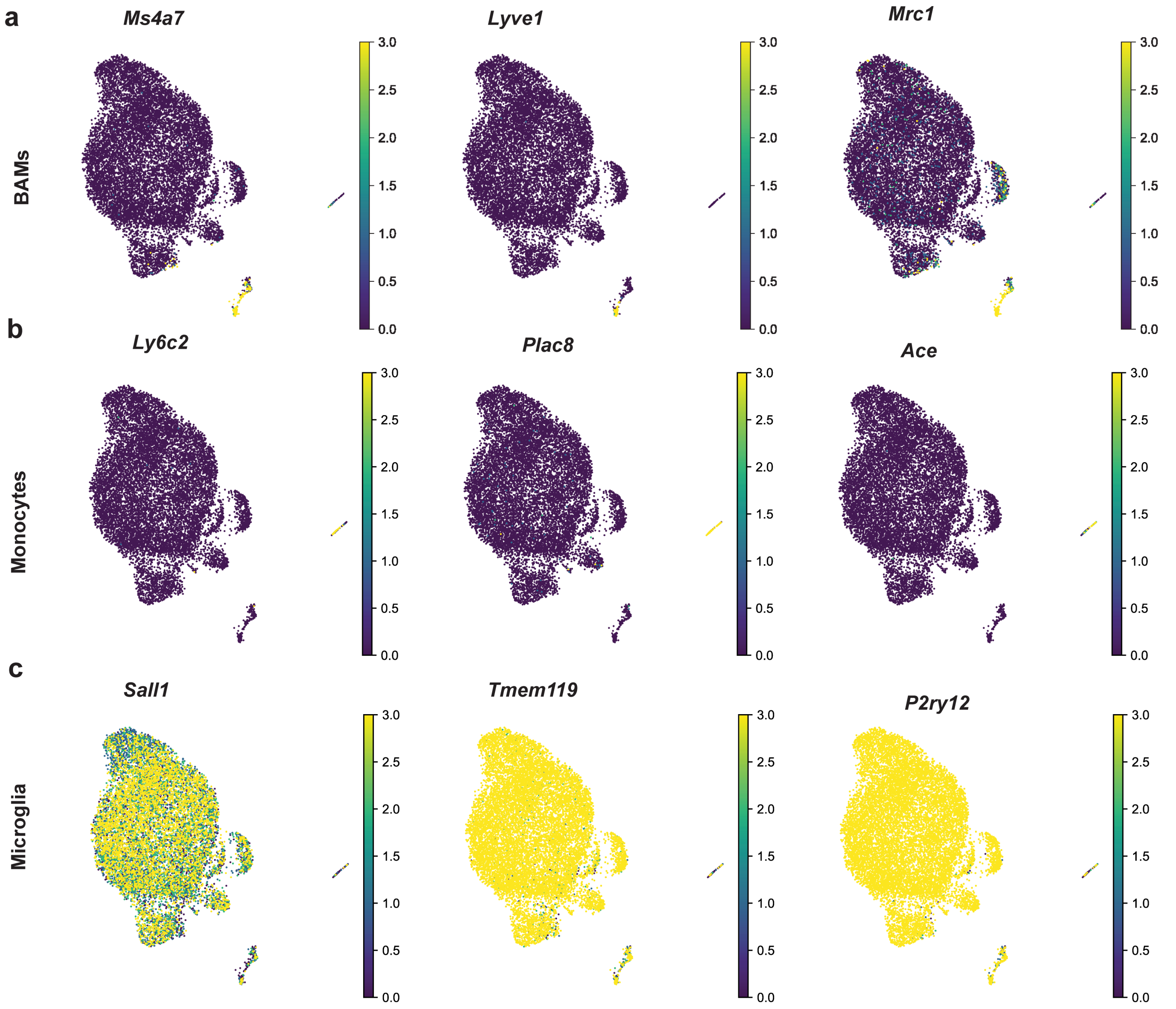

Suppl Fig. 4. **Cell annotation for BAMs, monocytes and microglia.** (**a**) BAM specific gene markers, *Ms4a7, H2-Eb1* and *Mrc1* exclusively expressed in cluster 9. (**b**) *Ly6c2, Plac8* and *Ace*, genes representative of monocytes expressed in cluster 11. (**c**) Validation for the microglia specific genes, *Sall1, Tmem119* and *P2ry12* for clusters 0-8.

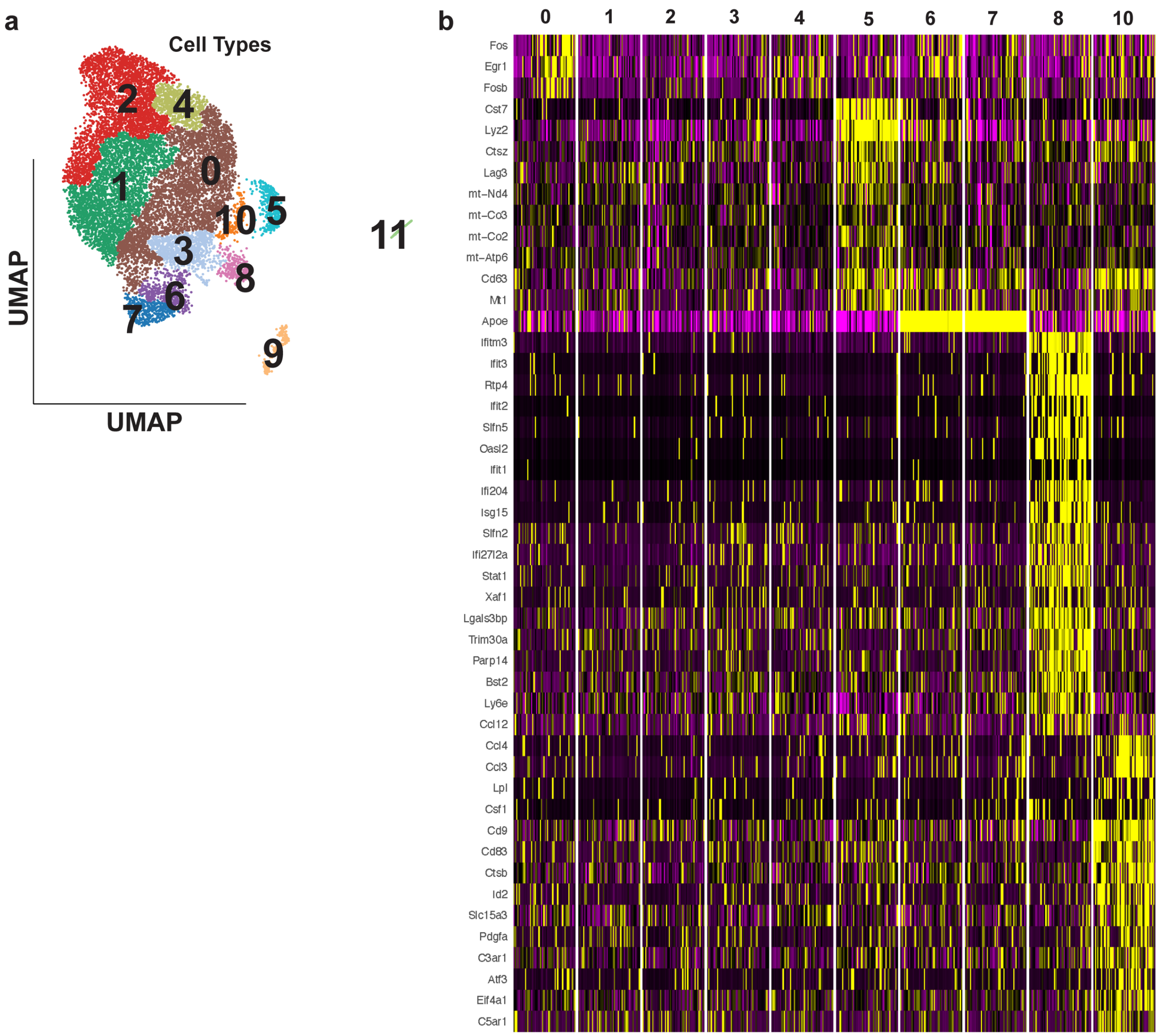

Suppl. Fig. 5. Differential gene expression of the microglia clusters from Experiment 1. (**a**) UMAP projection of the 12 identified clusters from Experiment 1. (**b**) Heatmap showing the top 50 differentially expressed genes in each cluster.

names	logfoldchanges	pvals_adj
Fos	3.978061	0.0
Egr1	3.7204356	1.57828414955067e-293
Jun	7.425133	1.12387293745217e-287
Jund	3.7566464	9.9211537417647e-234
Btg2	5.274881	3.47933159807078e-225
Fosb	2.7106755	8.78232032888286e-194
Junb	2.8097525	9.78255554752658e-179
Zfp36	1.7631305	4.13343065526503e-149
H3f3b	3.9211273	3.05017224701901e-121
Klf2	2.1629684	1.97961634434867e-98
Rps29	3.3274794	8.39300977492759e-95
Rhob	1.8594074	1.86124982023195e-90
Dusp1	1.7732257	5.70563761112089e-89
Ier2	1.6487031	3.8111038241236e-87
Btg1	1.6158478	1.68246859533707e-86
Zfp36l1	2.4676569	1.84055650695929e-85
Rps9	1.4756492	4.27764413514599e-84
Ubc	1.4073681	7.81378626388905e-76
Ddx5	1.914945	5.08386462427705e-75
Rpl39	1.3552799	1.42571703796906e-72
Rplp1	2.5459473	6.18059625100649e-67
Rps12	1.4597511	1.44439915727024e-65
Ubb	1.4266163	1.56819453491745e-64
Klf6	1.1965294	4.2732669976866e-63
Ptma	1.3182813	5.63474557203214e-63
Rps11	1.080711	1.04573220629386e-60
Rgs2	1.5053098	2.08236035126791e-60
Rps15a	0.92488134	9.79623058899814e-59
Rps21	1.4182543	1.08170466752692e-58
Rplp2	0.9798966	2.46253383087915e-58

Suppl. Fig. 6. The top 30 DEGs in Cluster 0 from Experiment 1.

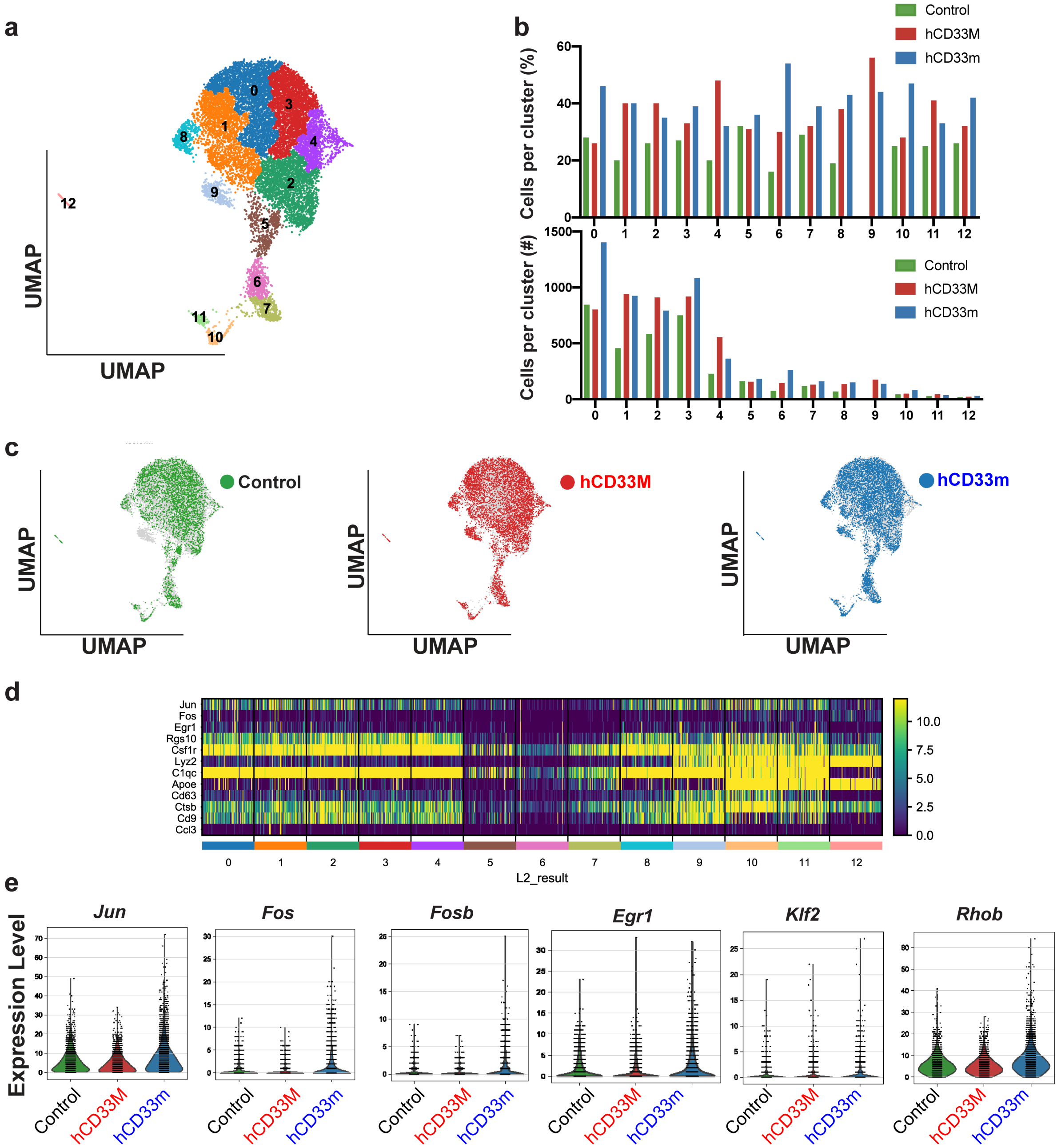

Suppl. Fig. 7. **Single cell analysis of control, hCD33M and hCD33m in Experiment 2 reveals differences in isoform gene expression.** (**a**) UMAP projections of the 13,982 cells in the merged Experiment 2 datasets showing 13 individual clusters. (**b**) Bar graphs showing the absolute number of cells from each isoform present in each cluster (top) and their respective proportions (bottom). (**c**) UMAP projection of the individual Control, hCD33M and hCD33m datasets. (**d**) Heatmap of representative genes. (**e**) Violin plots of hCD33m specific cluster 0 genes.

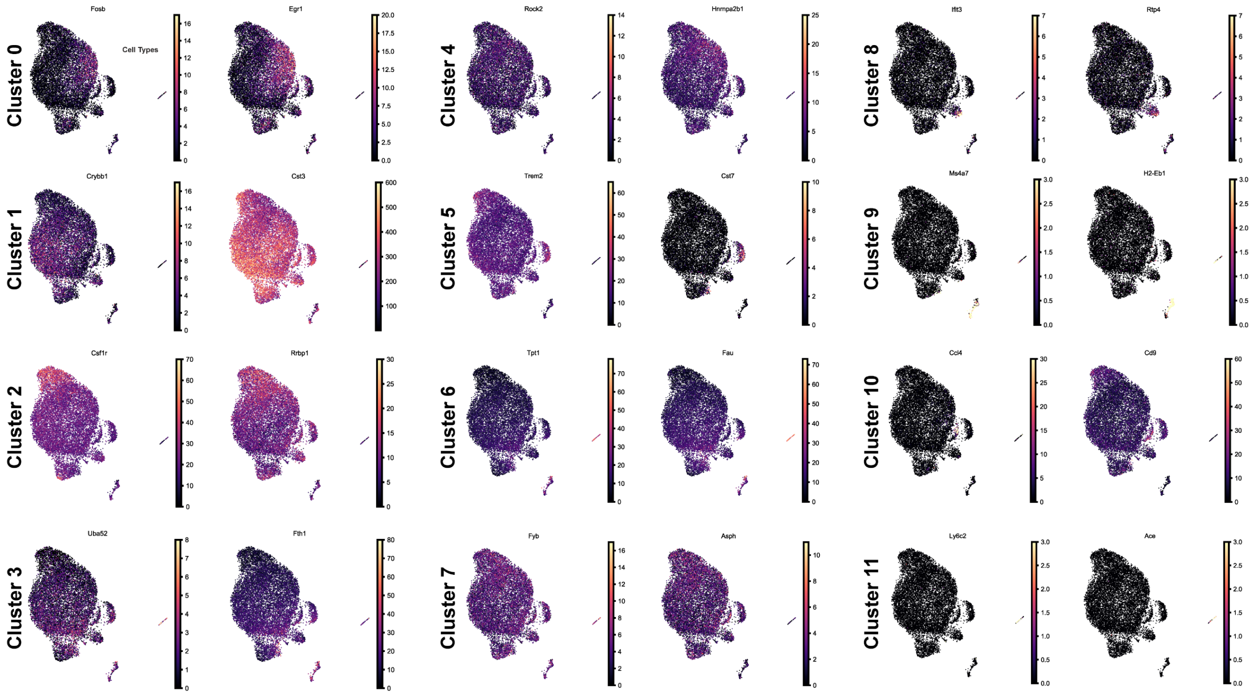

Suppl. Fig. 8. Feature plots showing the differentially expressed genes for each of the 11 clusters. Cluster were defined by the unsupervised SCCAF clustering and the expression of two representative genes were chosen for each cluster. Cluster 0-8, and 10 expressed microglial genes, whereas cluster 9 expressed border associated macrophage genes and cluster 11 expressed monocyte genes.

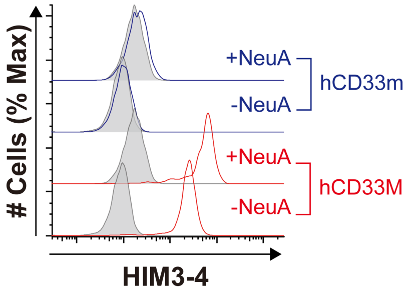

Suppl. Fig. 9. Anti-CD33 clone HIM3-4 does not recognize hCD33m. U937 cells overexpressing either hCD33M or hCD33m tested with anti-CD33 antibody clone HIM3-4 before and after pre-treatment with neuraminidase.

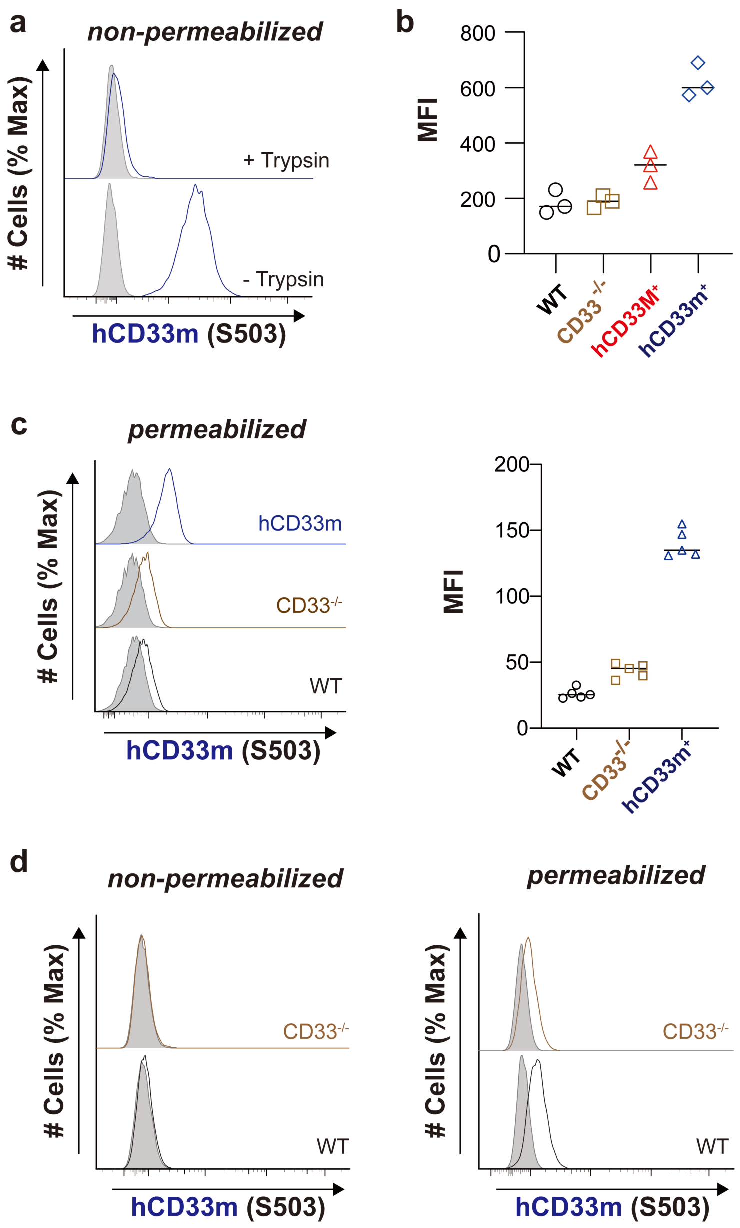

Suppl. Fig. 10. Optimizing and quantifying intracellular staining with S503 on U937 and THP1 cells. (a) CD33$^{-/-}$ U937 cells overexpressing CD33m were used to optimize a procedure with trypsin to remove cell surface antigens. Cells were treated with or without trypsin prior to staining with S503 (blue) or isotype control (grey). Cells were not fixed or permeabilized in this experiment. **(b)** Quantification of the mean fluorescence intensity (MFI) values for S503 staining of U937 cells with the indicated genotypes, taken from Fig. 5e of the main manuscript. MFI values are isotype control-subtracted. **(c)** An independent experiment showing that intracellular staining of hCD33m can be detected within hCD33m-overexpressing CD33$^{-/-}$ U937 cells. **(d)** Extracellular (*left panel*) and intracellular (*right panel*) staining with S503 and isotype control (grey) on WT and CD33$^{-/-}$ THP1 cells.

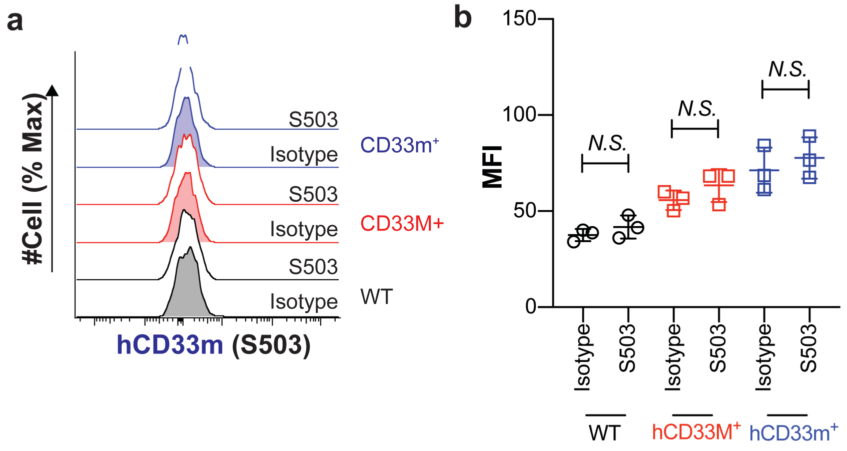

Suppl. Fig. 11. Cell surface staining of hCD33m$^+$ and hCD33M$^+$ transgenic primary mouse microglia with antibody S503. (a) Flow cytometry histograms of WT (black), hCD33M$^+$ (red), and hCD33m$^+$ (blue) primary microglia stanine with either isotype or S503. **(b)** Quantification of the mean fluorescence intensity (MFI) values for n=3 samples for each condition. *N.S.* = no statistical significance (P>0.05).

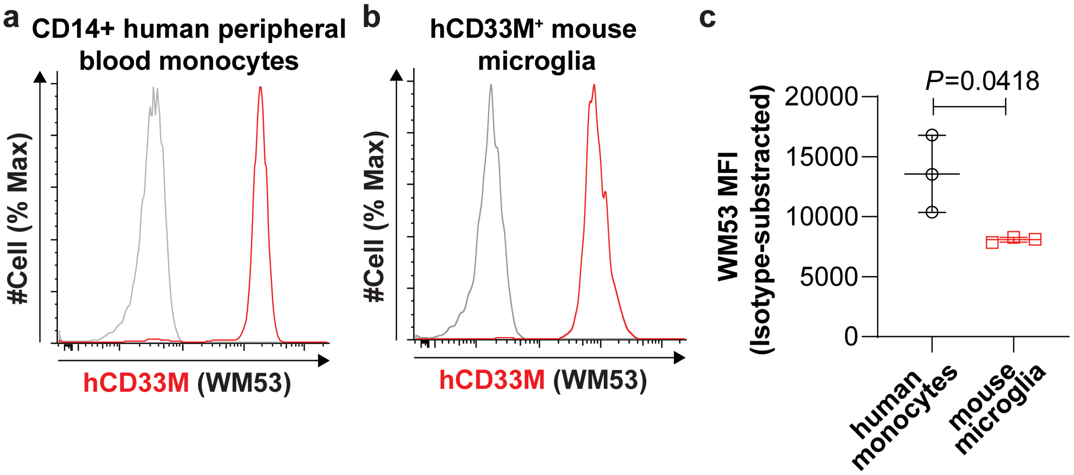

Suppl. Fig. 12. hCD33M+ mouse microglia express hCD33M at levels modestly lower than human peripheral blood monocytes. (a) Peripheral blood mononuclear cells were assessed for CD33M expression (red) on CD14+ peripheral blood monocytes relative to an isotype control (grey). **(b)** hCD33M+ primary mouse microglia were assessed for CD33M expression (red) relative to an isotype control (grey). **(c)** Mean fluorescence intensity (MFI) values from the flow cytometry graphs of CD33M signal. Data represents three different healthy human subjects and three different CD33M+ mice.

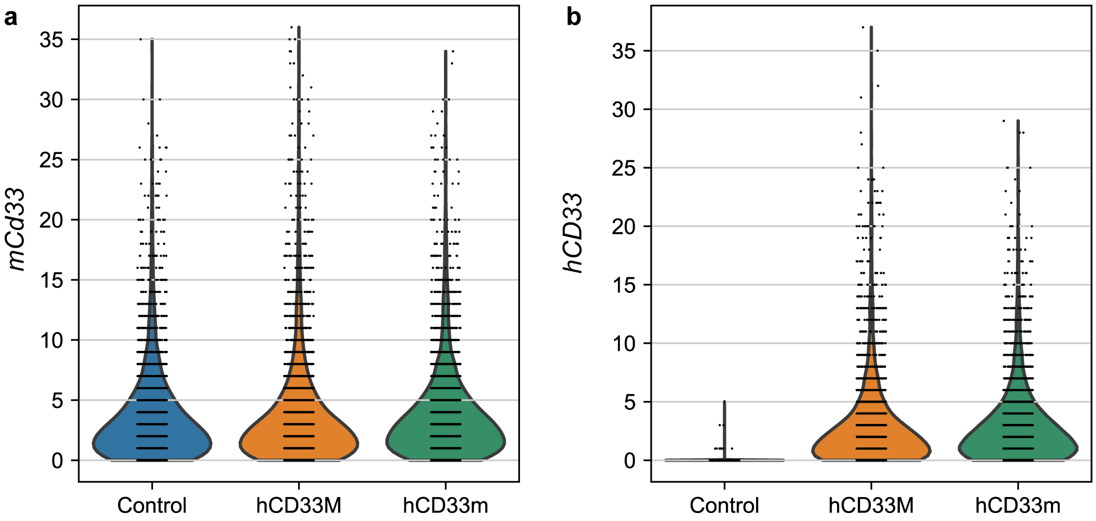

Suppl. Fig. 13. CD33 transcript levels in transgenic mouse microglia. (a) *mCd33* transcript levels in primary microglia from hCD33 transgenic mice demonstrate that expression of neither hCD33 isoform alters the *mCd33* transcript levels. **(b)** *hCD33* transcript levels in primary microglia from hCD33 transgenic mice. Both datasets are derived from aligning our scRNAseq datasets with the inclusion of *hCD33*. Note that this method does not differentiate *hCD33M* and *hCD33m* transcripts due to extensive overlap between the two isoforms.
